# Physiological, biochemical and molecular responses associated with drought tolerance in grafted grapevine

**DOI:** 10.1186/s12870-023-04109-x

**Published:** 2023-02-23

**Authors:** Shuzhen Jiao, Fanwei Zeng, Yaping Huang, Libing Zhang, Juan Mao, Baihong Chen

**Affiliations:** 1 College of Horticulture, Gansu Agricultural University, Lanzhou, 730070 Gansu China; 2 School of Agriculture, Ningxia University, Yinchuan, 750021 Ningxia China

**Keywords:** Grapevine, Grafting, Drought stress, Physiological and biochemical traits, Gene expression

## Abstract

**Background:**

Grafting is one of the promising techniques for improving abiotic stress tolerance in horticultural crops, but the underlying regulatory mechanisms of drought on grafted grapevine are largely unexplored.

**Results:**

Herein, we investigated the phenotypic, physiologic, biochemical, and drought related genes change of self-rooted 1103P (1103 Paulsen), SM (Shine Muscat) and grafted SM/1103P (SM shoot/1103P root) under drought stress condition. The results indicated that grafted grapevine effectively alleviated drought damage in grape leaves by higher RWC, water potential and free water content. Drought stress led to the alterations of chlorophyll, carotenoid, photosynthetic parameters and chlorophyll fluorescence in grapevine leaves after drought treatment indicated grafted plants improved the photosystem response to drought stress. Moreover, grafted plants under drought stress exhibited higher levels of abscisic acid (ABA), indoleacetic acid (IAA) and soluble protein, but less contents of hydrogen peroxide (H_2_O_2_) and malondialdehyde (MDA) both in leaves and roots. Drought stress also increased the activities of antioxidant enzymes (SOD, POD and CAT) and activated the transcript expression of *VvCu/ZnSOD*, *VvPOD4* and *VvCAT1*) in both leaves and roots. Further expression analysis by real-time PCR indicated that the expression levels of ABA-dependent and ABA-independent related genes could be activated in grafted grape after drought treatment.

**Conclusions:**

Taken together, our findings demonstrated that grafting onto 1103P enhanced tolerance against drought stress in grape by improving water content, photosynthesis and antioxidant defense capacity, which provided a valuable information for understanding the mechanisms of drought tolerance regulated by grafting plants.

**Supplementary Information:**

The online version contains supplementary material available at 10.1186/s12870-023-04109-x.

## Background

Drought is a misfortune for agriculture, which restricted plants growth, development and productivity throughout the world [1–3]. In order to minimize the effects of stress, plants have evolved various adaptive responses to maintain growth and productivity [4]. This involves a range of morphological, physiological and biochemical response in plants. For example, drought stress often caused grape leaves wilted and yellowed [5]. Plant responded to drought stress also by closing their stomata and accumulating compatible solutes to maintain a lower water potential [4]. Chlorophyll content was remarkably decreased under drought stress condition during plant growth [6]. Moreover, drought stress directly influences the photosynthetic rate and leaf gas exchange, which is co-related with hampered growth in plants [7]. Drought stress often promoted the production of intracellular reactive oxygen species (ROS), which might lead to membrane lipid peroxidation, chlorophyll bleaching, enzyme dysfunction, and protein oxidation and aggregation [8–10]. Then ROS can be scavenged through a series of antioxidant enzymes, such as superoxide dismutase (SOD), peroxidases (POD), and catalase (CAT) [11–13]. In response to drought condition, plants accumulate abscisic acid (ABA) to regulate responses to dehydration and optimize water use [3, 14]. Additionally, ABA controlled a diverse range of cellular and molecular processes and played critical roles in gene regulation, stomata closing, seed maturation, and dormancy after drought stress [15, 16].

Grafting is a very ancient method widely used in modern production of many horticultural plants for diverse purposes, such as clonal propagation, providing resistance to soil pathogens, enhancing the resistance to abiotic stresses and improving the yield and quality of horticultural plants [17–20]. In viticulture, grafting was almost imperative in Europe to control infestation by phylloxera diseases, a soil-dwelling insect pest introduced to Europe and gradually destroyed European vineyards [18, 21]. In parallel, rootstocks are selected in grafting grapevine for their influence on scion growth, fruit composition, and drought resistance [22]. Previous studies have shown that rootstock can significantly impact on the fruit composition, such as fruit berry size, yield, and quality parameters [23]. Some rootstocks can increase vegetative and reproductive growth. For example, a greater leaf area and higher yields were observed when sultana (*vitis vinifera* L.) grafted onto the rootstock of 41B [24]. Several studies reported an effect of rootstock on anthocyanin, glycosylate contents, and titratable acidity [25, 26]. The selection of rootstock also confer tolerance to the scion, especially increasing drought stress and improving water use efficiency [27, 28]. In grapevine (*Vitis vinifera* L.), the drought tolerant rootstocks could reduce the effect of water constraints by improving water uptake and transport and controlling the transpiration [28–30]. The research showed that SO4 (*Vitis berlandieri* × *Vitis riparia*) maintained higher stem water potential and net CO2 assimilation rate when compared with Cabernet Sauvignon grafted onto 1103P (1103 Paulsen) [31]. In addition, a large number of studies have investigated that rootstocks have the ability to influence the hydraulic and hormonal signaling pathways [22]. Several studies reported that hydraulic signals could induce leaf-derived ABA stomatal closure [32, 33]. Studies have demonstrated that drought resistance were different among the rootstock genotypes [34, 35]. For example, the 1103P, 140 Ruggeri, Kober 5BB and Richter 110 showed higher drought tolerance compared with the 101–14 and Schwarzmann [26]. Therefore, rootstock management is considered as an important way to improve the resistance of grapes to water shortage [36].

Grapevine is an important economic fruit crop which widely cultivated all over the world, and most of the world’s emerging grape-growing regions are located in arid or semi-arid regions, drought is a major limiting factor which seriously affects grapevine production and quality [37]. Grafting has been used widely in agriculture and also promotes tolerance to a range of abiotic stresses, the mechanisms of drought on grafted grapevine is still limited. With the aim of studying the drought tolerance mechanism of grafted grapevine, we carried out a comparative study on self-rooted scion, self-rooted rootstock and grafted grape to evaluate their comprehensive phenotypic, physiologic, biochemical characteristics and gene expression, and compared their different physiologic responses and potential regulatory networks under well-watered and drought stress condition. The objective of our study was to unravel the mechanism of drought tolerance induced by grafting, which may be promoted potential application of grafting in grape and other fruit trees.

## Results

### Effects of drought stress on the morphology of grapevines

**Fig. 1:**
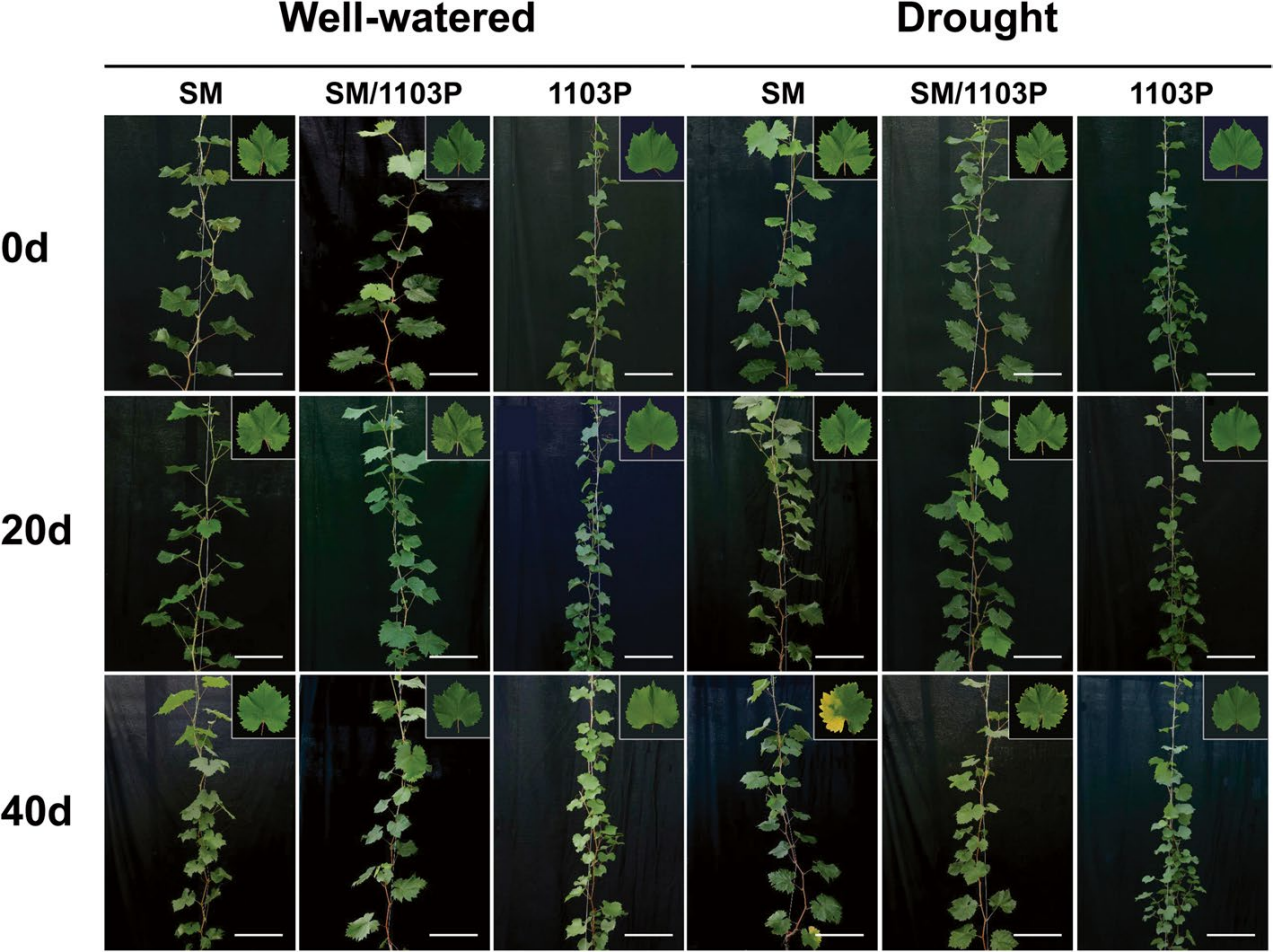
Growth performance of self-rooted and grafted vines under well-watered and drought condition. Highlighted in grey boxes were leaves with obvious signs. Well-watered: normal control, soil volumetric content was maintained at 60%; Drought: soil volumetric content was maintained at 20%. SM: self-rooted table grape cultivar ‘Shine Muscat’; SM/1103P: grafted grape (SM shoot / 1103P root); 1103P: self-rooted rootstock ‘1103 Paulsen’. Scale bars = 25 cm

The morphology of grapevine leaves were shown in Fig. 1, under well-watered condition, the leaves of three grape materials grew well and had no stress symptoms from 0 d to 40 d. On the 20th day of drought stress, the SM plants began to appear a few yellow spots on the edge of leaves, while the SM/1103P and 1103P had no leaf edge damage and remained healthily. At 40 d after treatment, the leaves of SM withered and the areas turned yellow, and some mature leaves had begun to curl. While the grafted plants of SM/1103P exhibited a less degree of leaf wilting and leaf edge damage, and the 1103P almost unaffected by drought stress, just like the well-watered groups. Thus, the grafted grapevine can reduce the drought damage compared with self-rooted SM.

### Effects of drought stress on the relative water content and water potential in grapevine leaves

**Fig. 2:**
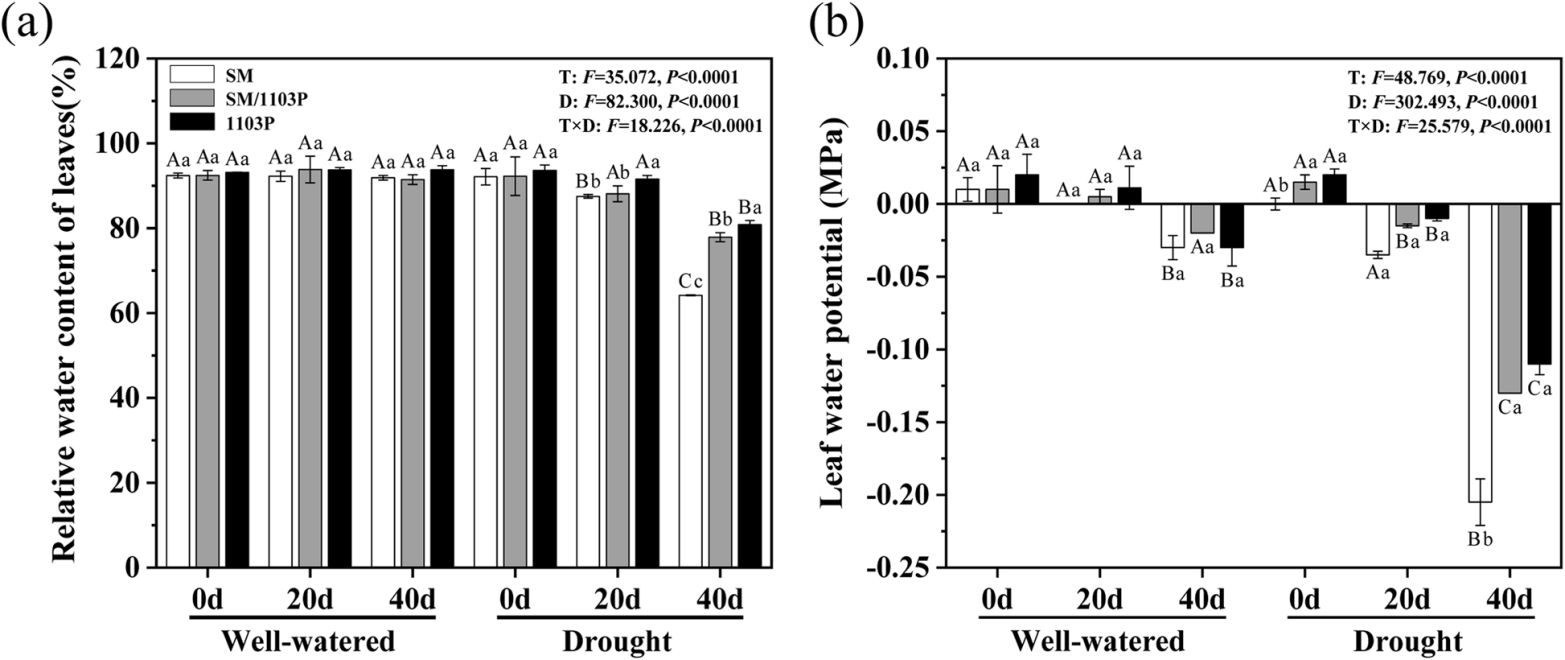
Relative water content and water potential of well-watered and drought stress in self-rooted and grafted vines leaves. **a** relative water content (RWC); **b** water potential (Ψ). The values are the means ± SD (*n* = 3). The “T” means three different grape materials in the same treatment period. “D” means the same grape material in different treatment periods, “F” means F-values. “T X D” indicates their interaction. Different lowercase letters denote significant differences among three different grape materials in the same treatment period, and uppercase letters represent significant differences in the same grape material at different treatment periods (*P < 0.05*) based on Duncan’s test

During plant development, drought stress significantly reduced RWC compared with the well-watered plants. RWC in three grapevine leaves were almost unchanged from 0 to 40 d in well-watered (91–93%), whereas the RWC kept highest in 1103P (80.8%), intermediate in SM/1103P (77.9%) and lowest in SM (64.2%) under drought stress, especially in 40 d after drought stress (Fig. 2a).

Leaf water potential (Ψ) in well-watered condition ranged − 0.03 ~ 0.02 MPa for the three grape materials, however the Ψ decreased gradually with ongoing of drought stress. Ψ was higher in 1103P than in SM/1103P and SM under drought stress in 20 and 40 d under drought treatment. The Ψ was − 0.01 and − 0.11 MPa in 1103P after 20 and 40 d of drought stress, − 0.015 and − 0.13 MPa in SM/1103P and the Ψ in self-rooted SM was − 0.035 and − 0.205 MPa, respectively, which indicated that grafting could alleviate the negative effect of drought stress compared to self-rooted SM (Fig. 2b).

### Effects of drought stress on the contents of total, free and bound water in grapevine leaves

**Fig. 3:**
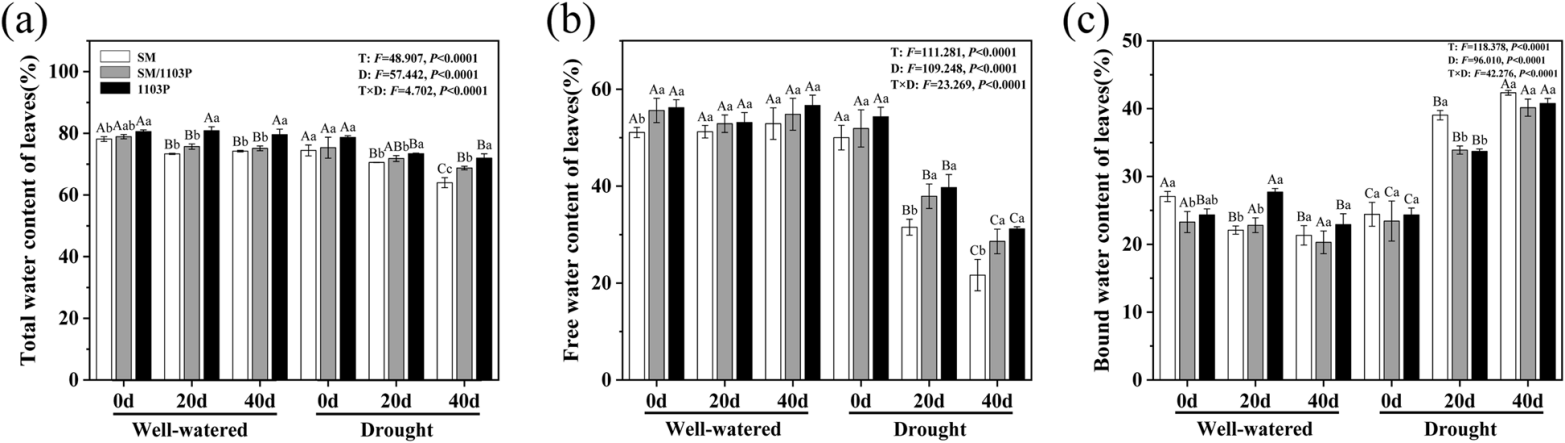
Total, free and bound water content of self-rooted and grafted vines in leaves under well-watered and drought condition. **a** total water content; **b** free water content; **c** bound water content. The values are the means ± SD (*n* = 3)

Drought stress could affect the total water contents of leaves compared with the well-watered condition. The total water contents decreased after drought stress. On the 20th day of drought stress, the total water contents of SM, SM/1103P and 1103P was 70, 71 and 73%, respectively. However, the total water contents reached a lowest degree on the 40th day of drought stress compared to the control condition (SM was 63%, SM/103P was 68% and 1103P was71%) (Fig. 3a). Leaf free water content and total water content showed a consistent trend, the free water contents of three grape materials decreased significantly at 20th day of drought stress (SM was 31%, SM/103P was 37% and 1103P was 39%). On the 40th day of drought stress, the free water contents of SM, SM/1103P and 1103P reached the lowest and decreased by 57, 44 and 42% compared the 0 d treatment (Fig. 3b). With the extension of drought stress duration, the content of bound water increased significantly, which was opposite to that of free water (Fig. 3c). Overall, different grape materials at same treatment level and the same grape plant on different treatment days and their interaction significantly affected the water physiological indexes (*P < 0.05*).

### Effects of drought stress on the contents of chlorophyll and carotenoid in grapevine leaves

**Fig. 4:**
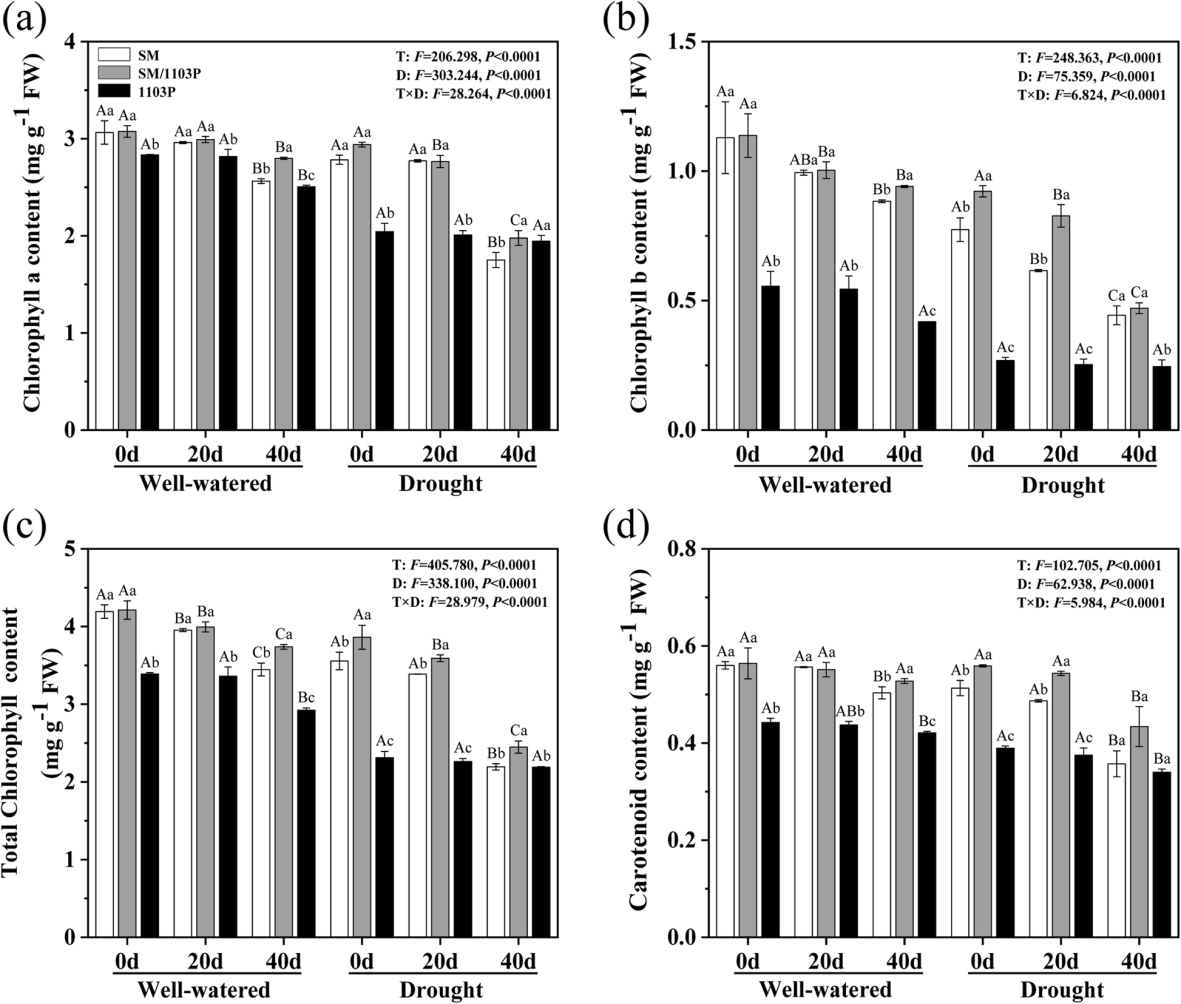
Chlorophyll and carotenoid contents of well-watered and drought stress in self-rooted and grafted vines leaves. **a** chlorophyll a content; **b** chlorophyll b content; **c** total chlorophyll content; **d** carotenoid content. The values are the means ± SD (*n* = 3)

The contents of chlorophyll and carotenoid declined gradually in self-rooted and grafted vines under drought condition, compared with that in the well-watered plants (Fig. 4). With the prolonging of drought treatment time, the contents of chlorophyll and carotenoid reduced, and showed obviously decreasing trend in 40 d after drought condition. Compared with SM, grafted SM/1103P showed higher amounts of chlorophyll (SM was 2.19 mg/g, SM/1103P was 2.45 mg/g) and carotenoid (SM was 0.36 mg/g, SM/1103P was 0.43 mg/g) after drought stress, yet the contents of chlorophyll and carotenoid in self-rooted rootstock 1103P (total chlorophyll was 2.18 mg/g, carotenoid was 0.34 mg/g) was lower than SM and SM/1103P. In addition, three grape plants, treatment days and their interaction significantly affected the content of Chlorophyll and Carotenoid (*P < 0.05*).

### Effects of drought stress on the photosynthetic parameters and chlorophyll fluorescence in grapevine leaves

**Fig. 5:**
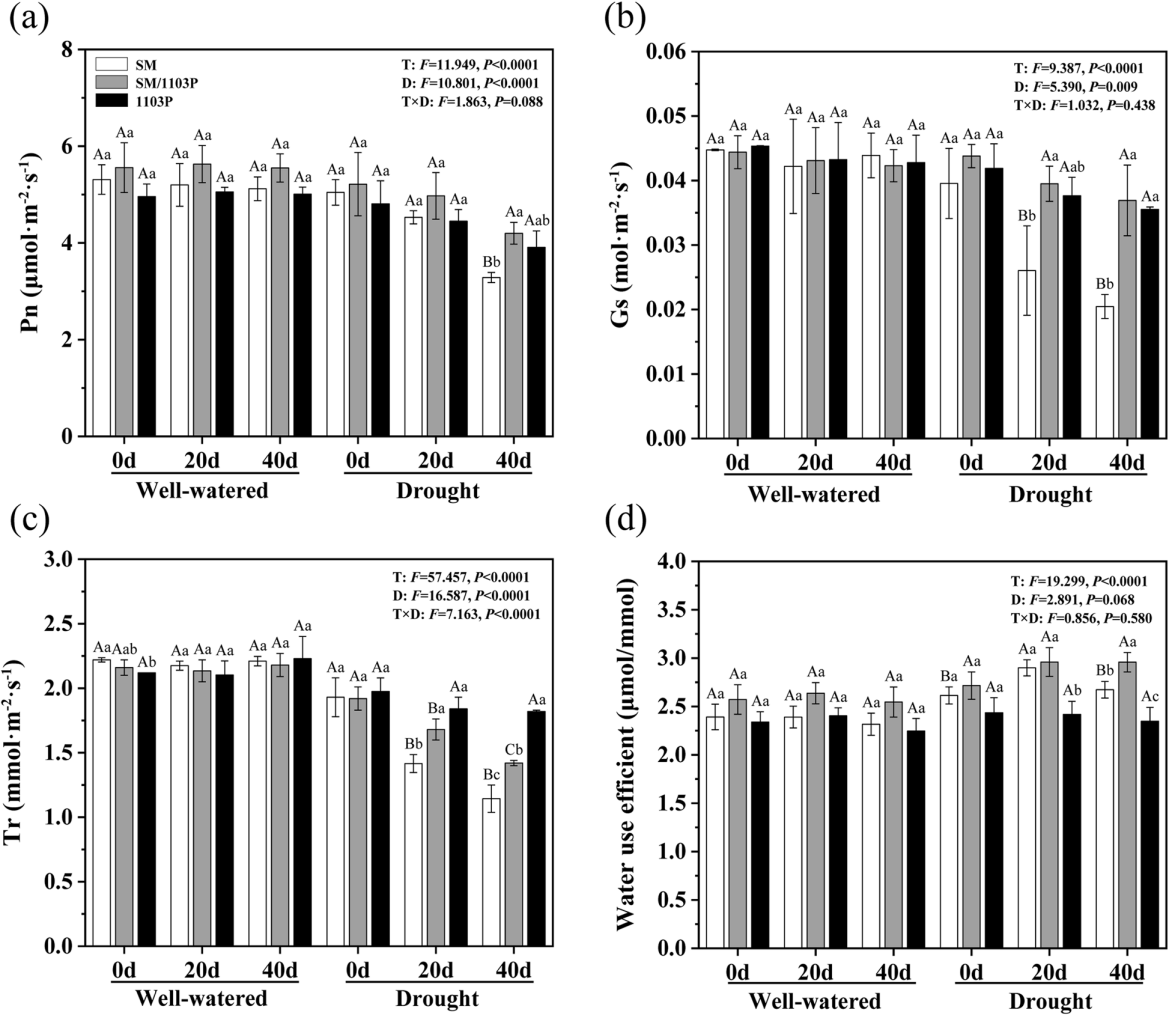
Effects of drought stress on photosynthetic parameters in self-rooted and grafted vine leaves. **a** net photosynthetic rate (Pn); **b** stomatal conductance (Gs); **c** transpiration rate (Tr); **d** water use efficiency. The data represent mean ± SD

For further verification, the photosynthetic parameters including Net photosynthetic rate (Pn), Stomatal conductance (Gs) and Transpiration rate (Tr) were measured in self-rooted and grafted grapevines. Under well-watered condition, there is not a sharp distinction of Pn among three grape materials. Pn decreased throughout the drought treatment, with values being significantly higher for grafted SM/1103P (4.2 μ mol·m^− 2^·s^− 1^) than that of the self-rooted SM (3.3 μ mol·m^− 2^·s^− 1^) at 40 d after drought stress (Fig. 5a). Values for Gs and Tr showed similar trends to Pn in the well-watered condition. However, values of Gs for SM (0.026 mol·m^− 2^·s^− 1^ for 20 d and 0.020 mol·m^− 2^·s^− 1^ for 40 d) showed a significant decrease compared to SM/1103P (0.039 mol·m^− 2^·s^− 1^ for 20 d and 0.037 mol·m^− 2^·s^− 1^ for 40 d) and 1103P (0.038 mol·m^− 2^·s^− 1^ for 20 d and 0.036 for 40 d) after drought stress (Fig. 5b). The values of Tr in drought condition were decrease in three materials, and exhibited higher in self-rooted 1103P (1.84 and 1.82 mmol·m^− 2^·s^− 1^ for 20 d and 40 d, respectively) and grafted SM/1103P (1.68 and 1.42 mmol·m^− 2^·s^− 1^ for 20 d and 40 d, respectively) than SM (1.42 and 1.14 mmol·m^− 2^·s^− 1^ for 20 d and 40 d, respectively) (Fig. 5c). Water use efficiency was higher in grafted vines than SM, which indicated that the decrease in transpiration rate was less than that of photosynthesis (Fig. 5d).

**Fig. 6:**
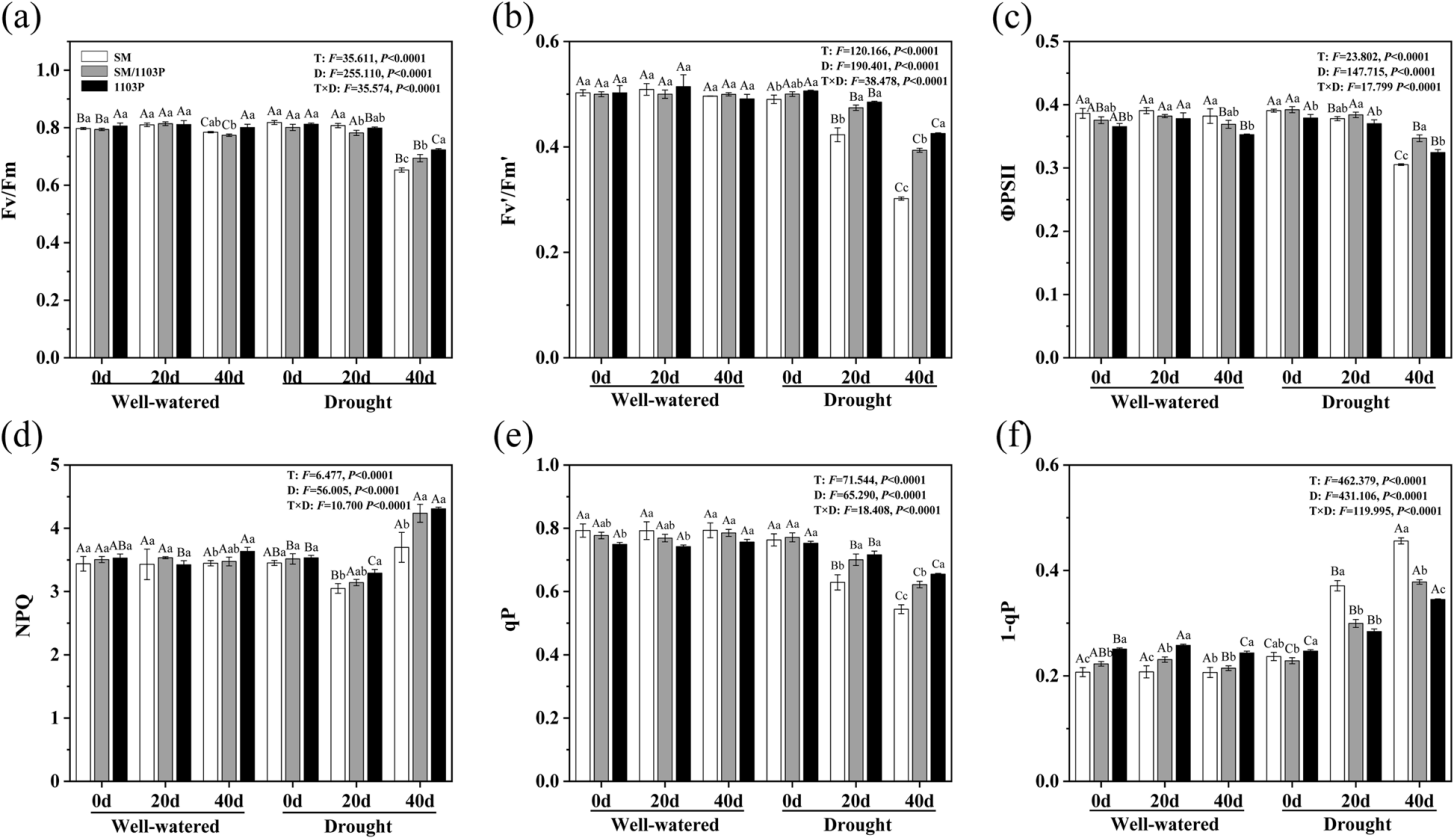
Effects of drought stress on chlorophyll fluorescence in self-rooted and grafted vine leaves. **a** maximum quantum efficiency of PSII photochemistry (Fv/Fm); **b** excitation energy capture efficiency of open PSII reaction centers under light (Fv’/Fm′); **c** the quantum yield of PSII (ΦPSII); **d** non-photochemical quenching (NPQ); **e** the photochemical quenching coefficient (qP); **f** Excitation pressure of PSII(1-qP). The data represent mean ± SD

There was no significant difference in Fv/Fm, Fv’/Fm′, ΦPSII and NPQ under well-watered condition. After drought stress, the Fv/Fm, Fv’/Fm′, ΦPSII and qP of the grape leaves continuously decreased, and a lower values were observed in SM than SM/1103P and 1103P (Fig. 6a-c and e). NPQ of grape leaves exhibited a slight decrease at 20d and a strong increase at 40 d under drought treatment (Fig. 6d). The excitation pressure (1-qp) of PSΠ was increased gradually with the stress time, while the rate of this increase in the self-rooted 1103P and grafted vines were lower than SM (Fig. 6f). Therefore, Grafted plants could inhibit the excitation pressure of PSII and reduce excess energy.

### Effects of drought stress on phytohormone contents in grapevine leaves and roots

**Fig. 7:**
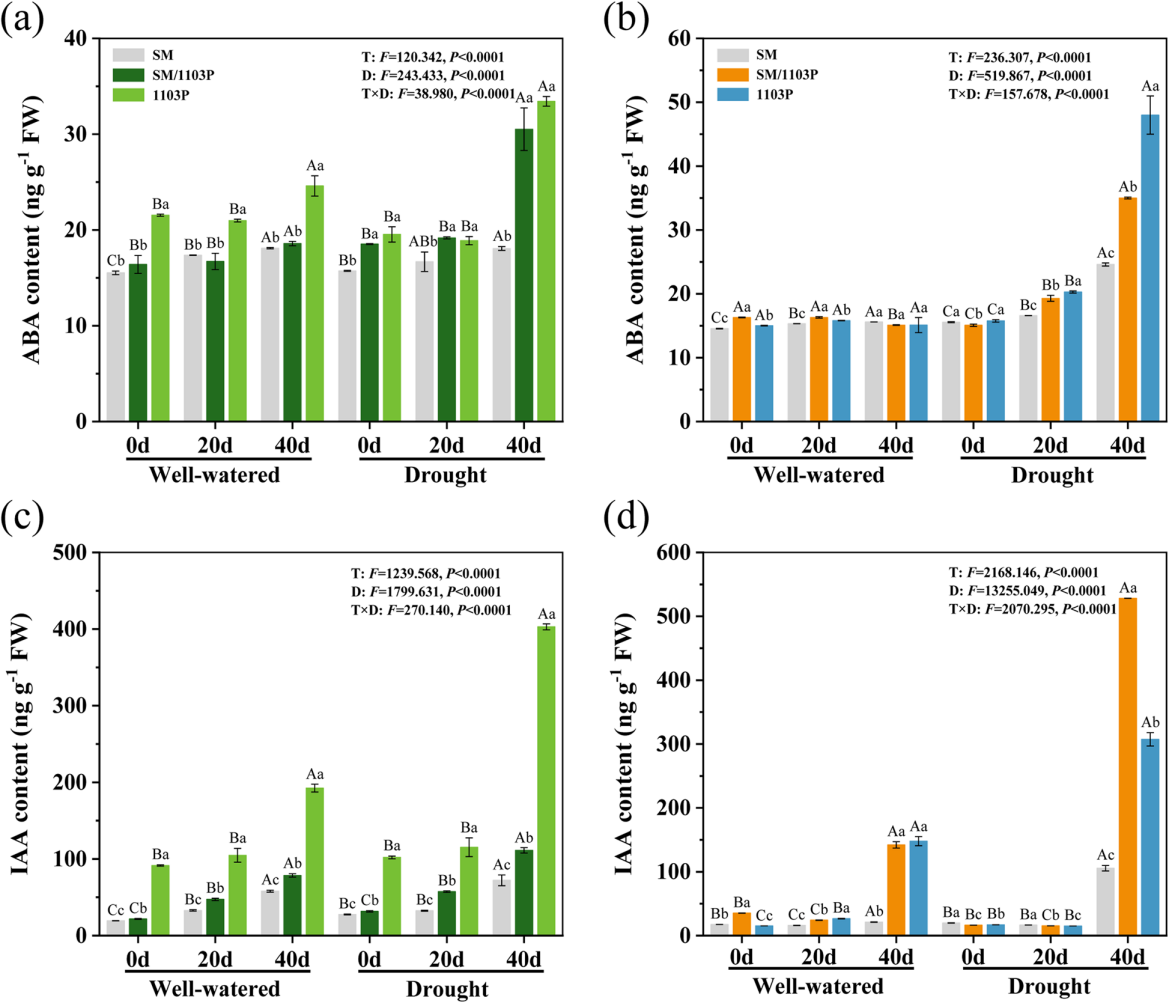
ABA and IAA contents in self-rooted and grafted vines under well-watered and drought condition. **a**, **c** quantification of ABA content (**a**) and IAA content (**c**) on leaves from SM,1103P and SM/1103P vines; **b**, **d** quantification of ABA content (**b**) and IAA content (**d**) on roots from SM,1103P and SM/1103P vines. The data represent mean ± SD

The endogenous hormone levels were analyzed in three grapevine leaves and roots (Fig. 7). Under well-watered condition, no significant difference of ABA and indoleacetic acid (IAA) concentrations were observed in leaves, yet the values were increased significantly with drought stress time. Compared to the 1103P (approximately increased by 42 and 75%, respectively) and SM/1103P (approximately increased by 39 and 71%, respectively), self-rooted SM (approximately increased by 13 and 62%, respectively) exhibited the less pronounced changes in ABA and IAA contents, especially in 40 d after drought stress (Fig. 7a and c). In the roots, there was no significant change of ABA content in well-watered condition, only slightly higher ABA levels were observed after 20 d stress, but high levels of ABA were found at 40 d after drought stress (Fig. 7b). The IAA levels in the root system remained virtually unchanged at 0 d and 20 d under control and drought condition. Whereas root IAA level at 40 d showed markedly changes, for example, under well-watered condition, root IAA levels were increased by 75% in SM/1103P and 89% in 1103P compared to 0 d. After drought stress, root IAA contents were increased dramatically in SM/1103P (approximately increased by 96%) and 1103P (approximately increased by 94%) (Fig. 7d). No significant increase values of root IAA were observed in SM on well-watered condition, but slight increase after drought stress. Thus, the results indicated that the accumulation of ABA and IAA increased drought tolerance of grapes. Furthermore, three grape plants, treatment days and their interaction significantly affected the leaves and roots phytohormone content *(P < 0.05)*.

### Effects of drought stress on the contents of H_2_O_2_, MDA and soluble protein

**Fig. 8:**
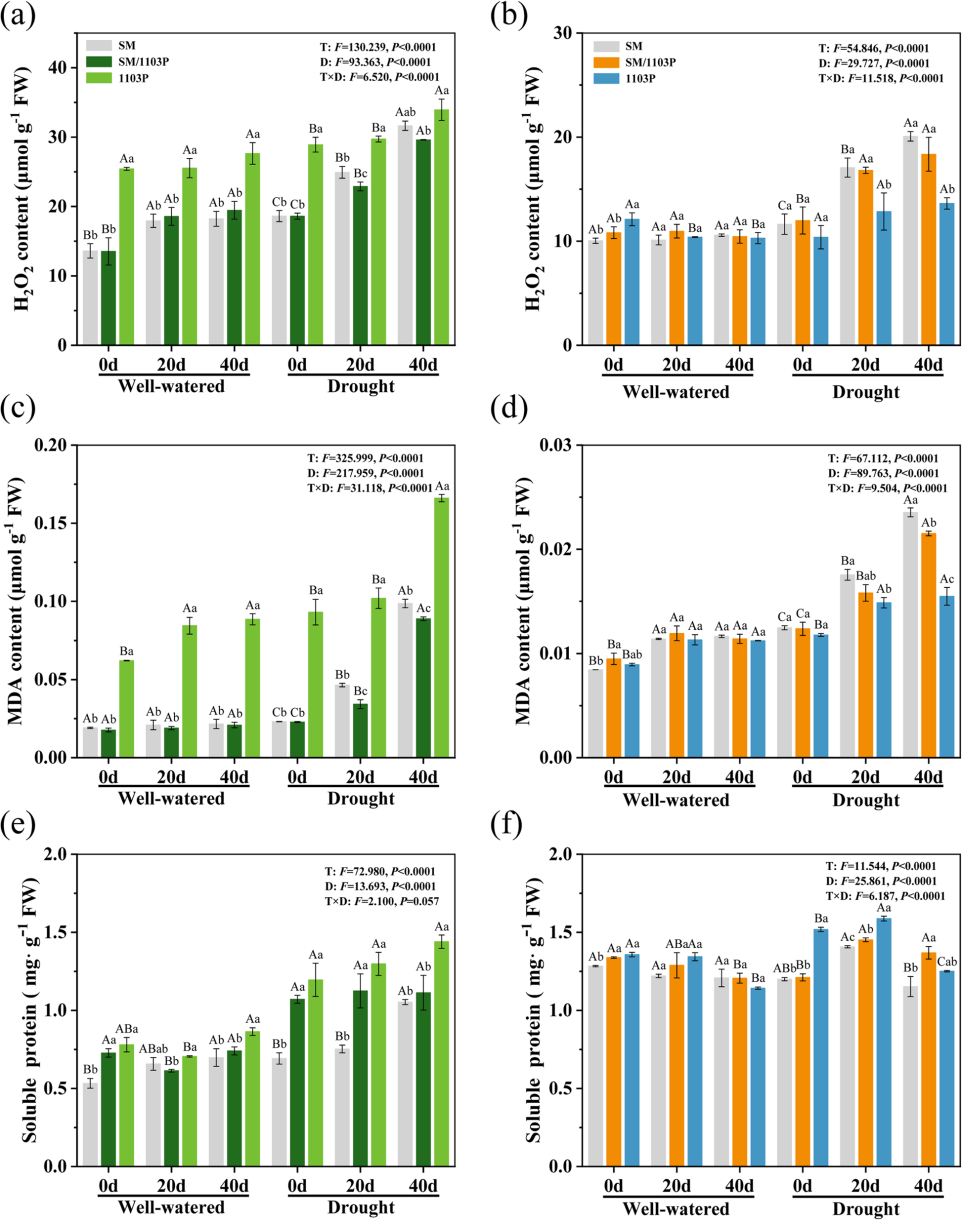
The contents of H_2_O_2_, MDA and soluble protein in self-rooted and grafted vines under well-watered and drought condition. **a**, **b** H_2_O_2_ contents on leaves (**a**) and roots (**b**) from SM,1103P and SM/1103P vines; **c**, **d**) MDA contents on leaves (**c**) and roots (**d**); **e**, **f** soluble protein on leaves (**e**) and roots (**f**). Each value is the mean ± SD of 3 replicates

To understand whether the grafting of SM/1103P could alleviate overproduction of lipid peroxidation and ROS in leaves and roots under drought condition, we investigated the contents of MDA and H_2_O_2_ among the three grape materials (Fig. 8). Under well-watered condition, the contents of H_2_O_2_ and MDA in leaves had no significant change from 0 d to 40 d. With the time-course extension of drought stress, the contents of H_2_O_2_ and MDA progressively increased in grapevine leaves, and the grafted SM/1103P (approximately increased by 37 and 74%, respectively) reduced the accumulation of H_2_O_2_ and MDA induced by stress compare with SM (approximately increased by 41 and 77%, respectively) (Fig. 8a and c). Root H_2_O_2_ and MDA levels showed a similar pattern with the time-course extension of well-watered condition. On the 20 d and 40 d of drought treatment, H_2_O_2_ and MDA obviously increased, and the contents in SM (approximately increased by 42 and 47%, respectively) were higher than SM/1103P (approximately increased by 35 and 43%, respectively) and 1103P (approximately increased by 23 and 24%, respectively) (Fig. 8b and d). These results imply that grafting may enhance drought tolerance by alleviating peroxidation of membrane lipids and reducing the accumulation of ROS.

Furthermore, quantitative analyses of soluble protein were conducted (Fig. 8e and f). Under well-watered condition, there was no significantly change between the grafted and self-rooted plants in leaves and roots from 0 d to 40 d. The soluble protein contents of leaves highly increased after drought stress. Compared with SM (0.69, 0.75, and 1.05 mg/g), the SM/1103P (1.07, 1.11, 1.12 mg/g) and 1103P (1.19, 1.30, 1.44 mg/g) had dramatically higher soluble protein contents (Fig. 8e). In roots, the soluble protein initially increased after 20 d treatment and then decreased after 40 d treatment. Meanwhile, the results showed that self-rooted SM had lower concentrations of soluble protein compared to SM/1103P and 1103P, indicating that grafting can enhance drought tolerance by increasing the contents of soluble protein (Fig. 8f).

### Effects of drought stress on antioxidant enzymes activities and transcriptional levels

**Fig. 9:**
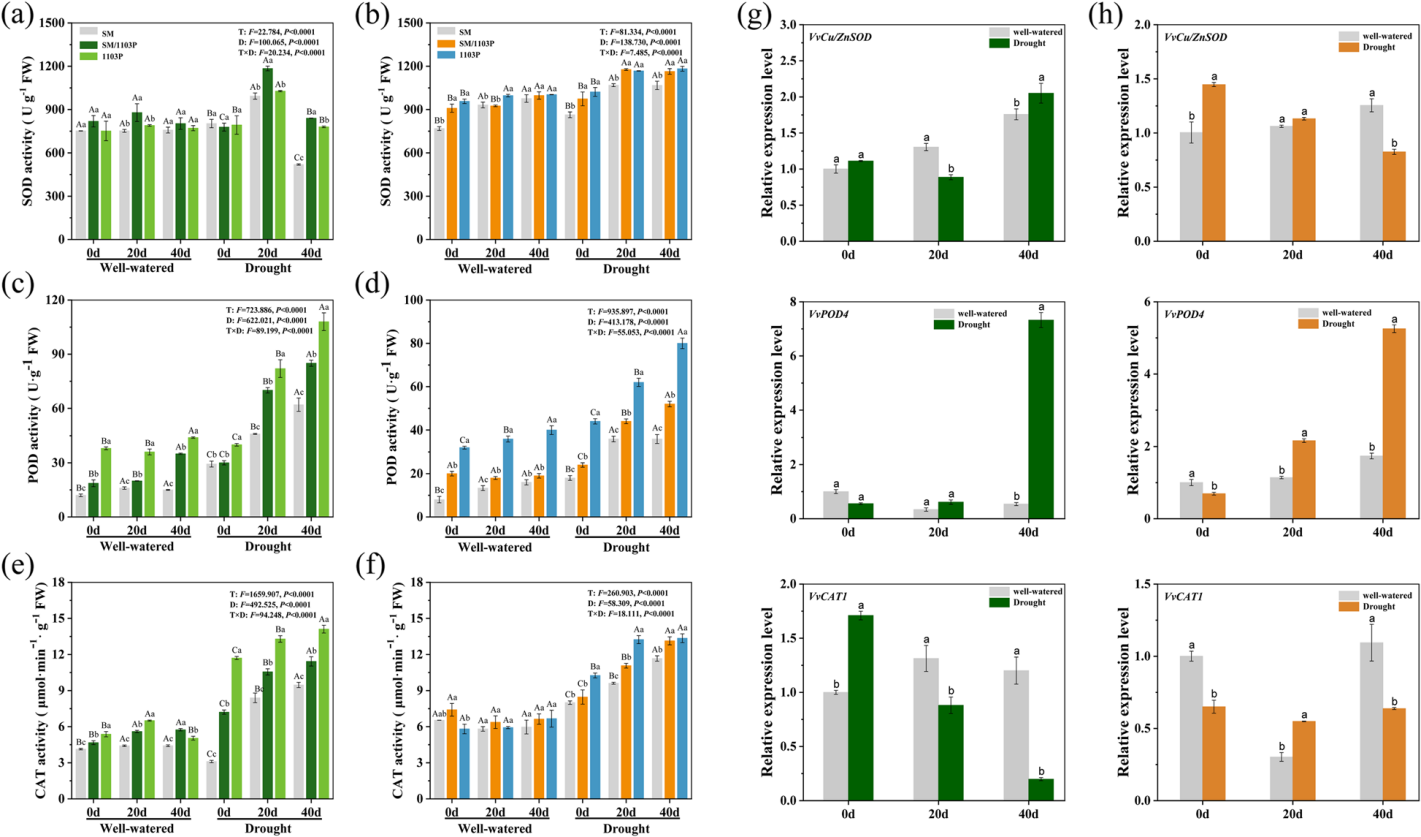
The enzymes activity and transcript expression levels of antioxidant enzymes in self-rooted and grafted vines under well-watered and drought condition. **a**, **b** SOD activity on leaves (**a**) and roots (**b**); **c**, **d** POD activity on leaves (**c**) and roots (**d**); **e**, **f** CAT activity on leaves (**e**) and roots (**f**) from SM,1103P and SM/1103P vines; **g**, **h** The expression levels of *VvCu/ZnSOD*,*VvPOD4* and *VvCAT1* in leavse (**g**) and roots (**h**). Data are average values of three replicates

We further examined the activity of antioxidant enzymes (SOD, CAT and POD) and evaluated the level of toxic oxidation products under drought stress (Fig. 9). No significant discrepancies were observed in SOD, POD and CAT activity between leaves and roots under well-watered condition. Under drought treatment condition, the activity of SOD in leaves increased significantly in 20 d stress and then fell prominently in 40 d stress. Root SOD activity increased steadily under drought stress in comparison to 0 d values (Fig. 9a and b). The activity of POD and CAT exhibited similar trends under drought stress, which increased dramatically both in leaves and roots (Fig. 9c-f). Moreover, the activity of the three enzymes in SM/1103P and 1103P were higher than that of in SM both in leaves and roots under drought stress. These results indicate that the rootstock positively affected the grafted plant by increasing the antioxidant enzymatic activity under drought stress. The three grape plants, treatment days and their interaction significantly affected the activity of antioxidant enzymes of leaves and roots overall *(P < 0.05)*.

The quantification of the relative expression levels of *VvCu/ZnSOD*, *VvPOD4* and *VvCAT1* were conducted in grafted grapevine to support qualitative analysis. As shown in Fig. 9g and h, compared with well-watered condition, the transcriptional levels of *VvCu/ZnSOD* in leaves were up-regulated in 0d and 40 d but downregulated in 20 d, whereas upregulated in roots in 0 d and 20 d. The *VvPOD4* expression levels in leaves and roots were upregulated in 20 d and 40 d stress, which showed a trend consistent with its corresponding enzyme activity. Meanwhile, compared with well-watered condition, the transcriptional level of *VvCAT1* in leaves was downregulated with the extension of drought stress duration, and up-regulated after 20 d stress in roots.

### Effects of drought stress on expression patterns of stress-responsive genes

**Fig. 10:**
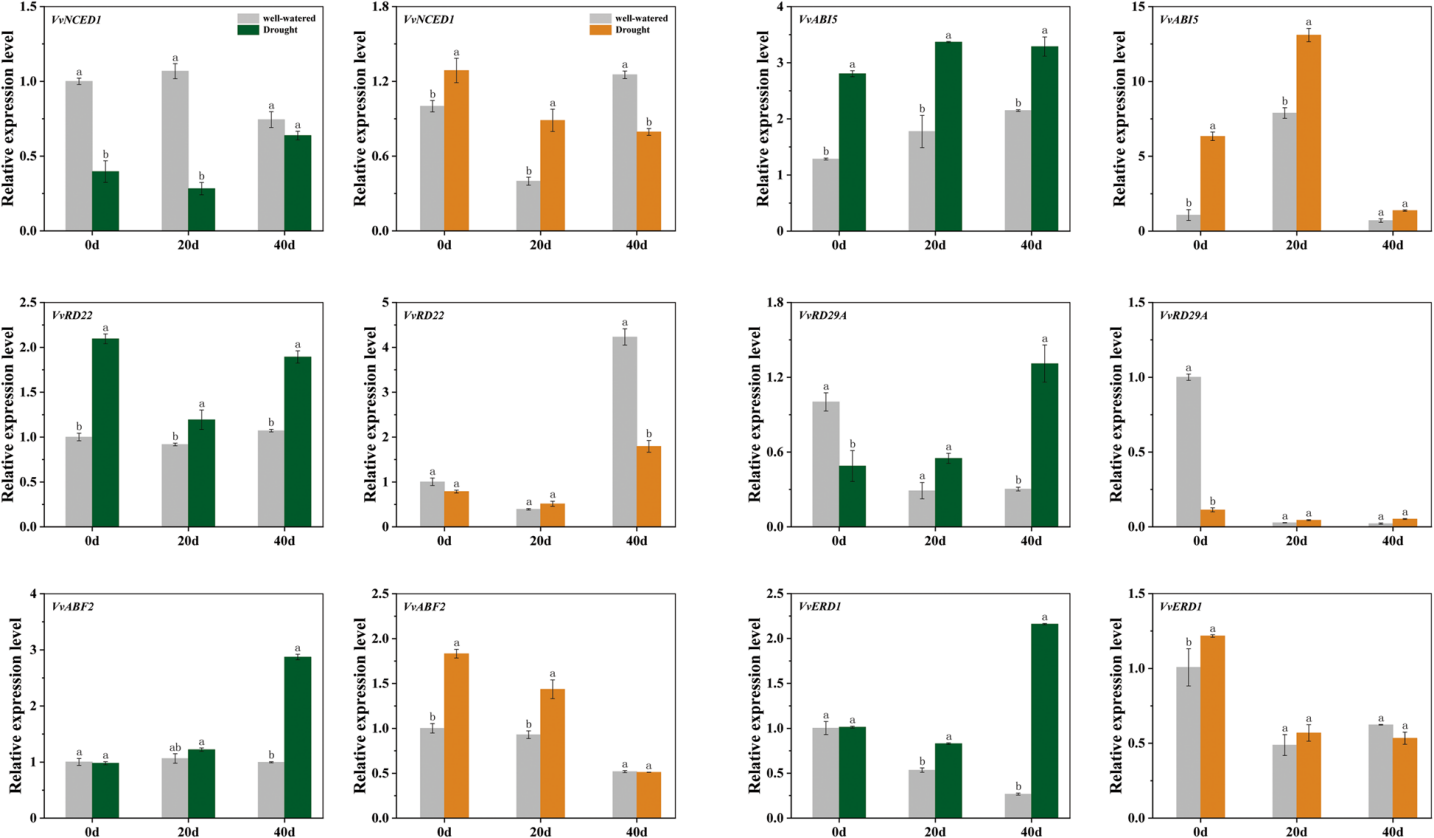
Transcript level of genes involved in ABA-dependent and ABA-independent signaling pathway in grapevine leaves (gray and green bars) and roots (gray and orange bars). Each value is the mean ± SD of 3 replicates, and lowercase letters denote significant differences (*P < 0.05*) based on Duncan’s test

To understand whether the grafted plant could alleviate drought stress are the results of gene regulation, qRT-PCR was used to detect and quantify mRNA transcript levels of stress-responsive related genes in leaves and roots (Fig. 10). The transcriptional levels of *NCED1* was downregulated in leaves but up-regulated in roots, compared to the well-watered condition. In leaves, compared with the well-watered condition, the expression levels of *VvABI5* (increased by 1.5–1.9 fold), *VvRD22* (increased by 1.3–1.8 fold), *VvRD29A* (increased by 1.9–4.3 fold)*, VvABF2* (increased by 1.1–2.9 fold), and *VvERD1* (increased by 1.6–8.1 fold) was up-regulated in 20 d and 40 d stress condition. In roots, *VvABI5*, *VvABF2* and *VvERD1* displayed higher transcript levels in 0 d and 20 d stress condition than in well-watered condition. In detail, *VvABI5* was increased by 1.7–5.9 fold, *VvABF2* and *VvERD1* was increased by 1.5–1.8 fold and 1.1–1.3 fold, respectively. The genes of *VvRD29A* and *VvRD22* in roots showed similar expression patterns, which was downregulated after 0 d drought stress.

### Principal component analysis (PCA)

**Fig. 11:**
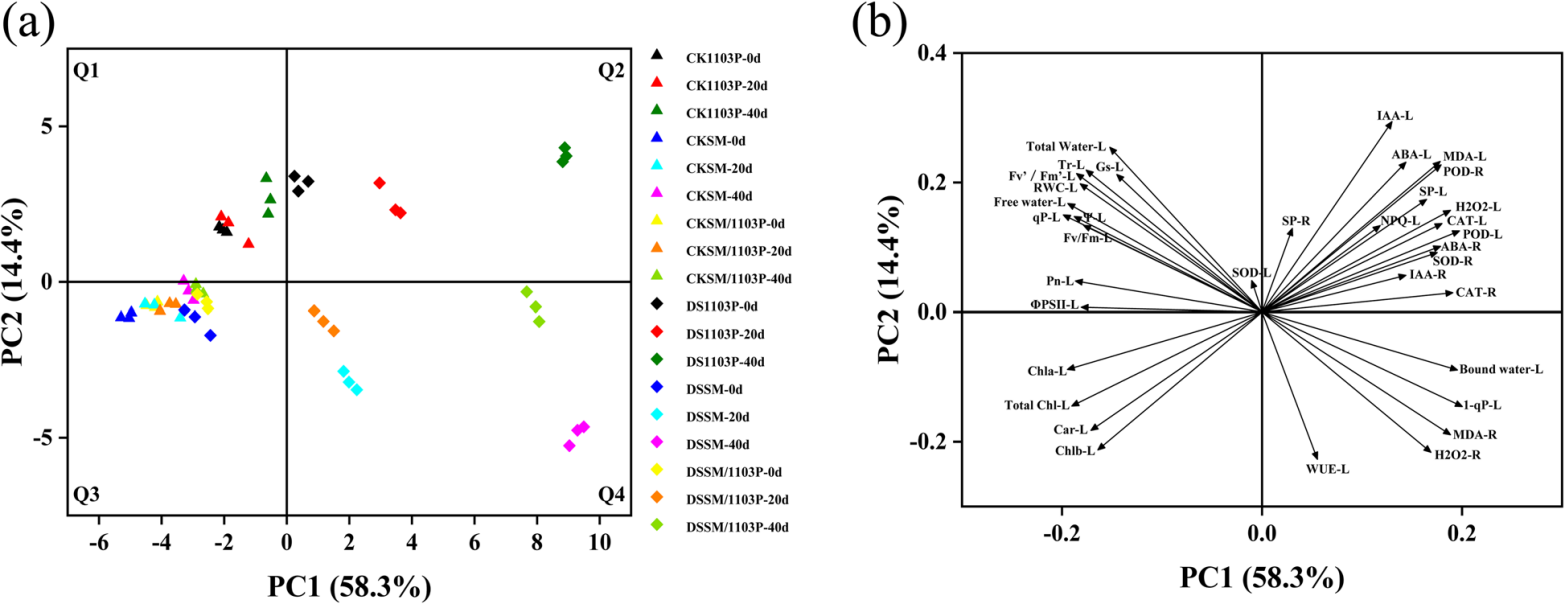
Principal component analysis of the internal quality parameters of self-rooted and grafted vines. **a** Score plot (**b**) loading plot. “△” represented well-watered samples and“◇” represented drought stress samples

In order to better visualize the internal parameters of self-rooted and grafted vines, PCA was carried out on the 27 compounds of grapevine leaves and roots (Fig. 11). The first two principle components (PCs) accounted for 72.7% of the total variation. The main component 1 accounted for 58.3% which was correlated positively with hormone (ABA and IAA), antioxidant enzyme (SOD, POD and CAT), MDA, H_2_O_2_ and soluble protein. Principle component 2 explaining 14.4% of the total variance was associated with photosynthetic parameters, chlorophyll fluorescence and water physiology related indexes (RWC, water potential, total and free water). Moreover, the well-watered (CK) samples locating in Q1 and Q3, separated from the drought stress (DS) samples by PC1.

Principle component analysis was independently performed for each grape material to further explore the difference of the internal parameters. The first component was 75.5% in SM, 74.4% in SM/1103P, and 66.7% in 1103P, respectively. They were associated with phytohormone (ABA and IAA), antioxidant enzyme (SOD, POD, CAT), MDA, H_2_O_2_ and soluble protein. Furthermore, the samples of drought stress on 20 d and 40 d were located in Q2 and Q4, and most of well-watered samples were located in Q3 (Supplementary Fig. 1).

## Discussion

In recent years, grafting has been used to protect against abiotic tress in many horticultural crops. The proper rootstock with tolerance to drought stress plays a crucial role to prevent water stress in many plant species [34, 38, 39]. However, there are relatively few studies evaluating the effects of rootstock on grafted vines and its possible mechanism under drought condition. In the current study, we investigated the effects of drought stress on self-rooted and grafted grapevines in phenotypic, physiologic, biochemical and molecular levels. These results provide valuable evidence involved in understanding the mechanisms of drought tolerance in grafting grapevine regulated by rootstock.

When plants are subjected to drought stress, some of grape leaves turned yellow and began to curl after 40 d treatment, while the grafted plants of SM/1103P and self-rooted 1103P exhibited less symptoms of drought, especially the 1103P group. Water status is an important physiological parameter and the RWC of plant leaves can reflect the level of drought resistance and water absorption efficiency [40, 41]. Our study showed a higher leaf RWC in grafted grape than in self-rooted SM after drought stress, indicating that the rootstock could help grafted plants to mitigate the loss of water and acquire higher water usage in the drought condition. The plant water potential has played an important role not only in determining plant response to drought stress but also influencing the metabolic processes of plants [42, 43]. In grapevine, the leaf water potential is considered one of the best-suited indicators of drought stress [44]. Our findings indicated that the self-rooted SM kept a lower level Ψ compared with grafted SM/1103P and self-rooted 1103P, which indicated that grafted grapevine have better resistance to drought. The contents of free and bound water can be used as physiological indicators to judge the drought resistance of plants. Under drought stress, the total water and free water contents showed a less decrease and bound water contents exhibited a less increase in SM/1103P and 1103P than that of SM, which indicated that tolerant grapevine could reduce water loss under drought condition. Chlorophyll is the main pigment in photosynthesis and grapevine leaves have a lower contents of chlorophyll due to the decrease in water content during the drought stress condition [45, 46]. The contents of chlorophyll declined progressively in both grafted and self-rooted plants after drought stress, but the grafted plants maintained higher levels throughout the drought treatment. Previous studies demonstrated that the functionality of photosystem was effected by drought stress [47]. Under drought stress, the photosynthesis rate reduces due to a decrease in the chlorophyll content of leaves and stomatal limitation [48, 49]. In the present study, Pn, Gs, Tr and water use efficiency of the grafted plants were higher than that of the SM, which could primarily be attributed to the drought-tolerant rootstocks of 1103P. The results of our study were consistent with previous analyses that grating onto drought-tolerant rootstocks could increase tolerance against drought, such as apple and chrysanthemum [50, 51]. As for the chlorophyll fluorescence (Fv/Fm, Fv’/Fm′, ΦPSII and qP), the grafted plants displayed a slowly decrease under drought stress condition. Our results indicated that grafting onto rootstocks could improve the drought tolerance of grape, it may be that stomatal or non-stomatal factors reduced the incidence of photosynthesis.

ABA is a major phytohormone regulating plant growth, development and response to dehydration [52]. It has an important ability to reduce water loss by inducing stomatal closure, improving antioxidant capacity, regulate photosynthesis and the stress response genes [53]. The accumulation of ABA is one of the key mechanisms of plant to adapt to drought stress [54, 55]. It has been found that drought stress caused greater accumulation of ABA both in roots and leaves in our research, especially in grafted plants and self-rooted 1103P, which indicated that the accumulation of ABA increased drought tolerance of grape. IAA is an important plant hormone and it regulates various aspects of plant growth and development, including apical dominance, tropic responses, lateral root formation, vascular differentiation, embryo patterning, and shoot elongation [56–58]. It has been found that grafted plants also had higher IAA content at 40 d under drought treatment. These results indicated that the increase of IAA improved the drought tolerance of grafted plants by mediating rootstock effects on shoot physiology [59]. We also observed the transcriptional levels of ABA-dependent related genes (*VvNCED1*, *VvABI5*, *VvABF2*, *VvRD22* and *VvRD29A*), which played an important role in ABA biosynthesis and signaling pathway. Consistent with previous studies in other plant species [60–63], the *VvABI5* and *VvABF2*, two basic leucine zipper (bZIP) transcriptions factor that functioned in ABA signaling and regulated many stress-responsive genes expression, were upregulated both in leaves and roots compared to well-watered condition. This further suggested that grafted plants can response to drought stress by inducing the expression of genes related to ABA signaling pathway. Concurrently, *VvRD22* and *VvRD29A,* the key genes of ABA signaling pathway, expression levels were increased significantly in leaves and decreased in roots after drought stress. The *VvNCED1* (9-cis-epoxycarotenoid dioxygenase 1), a key enzyme involved in ABA biosynthesis, was downregulated in leaves but upregulated in roots, indicating that there may be different expression patterns of these genes in leaves and roots under drought stress condition.

Drought stress negatively affects many aspects of cellular physiology, resulting in oxidative stress and the accumulation of reactive oxygen species (ROS), such as superoxide anion (O_2_^−^), hydrogen peroxide (H_2_O_2_) and hydroxyl radical (**·**OH) [64]. Excessive accumulation of ROS caused cell membrane damage, lipid peroxidation and DNA modifications, leading to cell death and loss of biomass [65]. Under drought condition, it has been shown that the content of H_2_O_2_ increased significantly in leaves and roots, but lower H_2_O_2_ contents were observed in grafted plants when compared with self-rooted SM, suggesting that grafted plants reduced oxidative stress and exhibited higher drought tolerance capacity. Furthermore, the contents of MDA are often used as the indicator for estimating membrane lipid peroxidation stress. In our study, the MDA contents were also increased after drought stress treatment but accumulated lower in grafted plants compared with SM, suggesting that grafted plants could alleviate membrane damage in response to drought stress. Similar reduction of MDA level was also observed in grafted chrysanthemum and tobacco [51, 66]. The contents of H_2_O_2_ and MDA were higher in 1103P compared with grafted SM/1103P and self-rooted SM under well-watered and drought condition, which might be caused by the thinner and tender leaves of 1103P.

To overcome the effects of oxidative stress and maintain redox homeostasis, plants invoke an antioxidant defense system, such as SOD, POD, CAT and other enzymes [64, 67]. SOD is the crucial antioxidant enzyme that thought to be played important roles in protecting the cells from cellular damage caused by ROS in the living cells [68]. Our study revealed that SOD activity was remarkable higher in grafted grape and self-rooted 1103P than self-rooted SM during the drought treatment. It worth noting that the levels of SOD activity in leaves were increased on 20 d but decreased on 40 d after drought stress, suggesting that the defense mechanism of the SOD may have been destroyed with the extension of stress time. This phenomenon was also reported in other species under drought stress [69–72]. POD and CAT are major antioxidant enzymes that can essentially scavenge the accumulated H_2_O_2_ to non-toxic levels by converting it into water and molecular oxygen, thus preventing cellular damage [73]. In the present study, POD and CAT activity increased under drought stress condition, while compared with the self-rooted SM, the level of CAT activity in grafted plants was remarkably higher. Interestingly, we found that the expression of *VvCu/ZnSOD* and *VvCAT1* were not always correlated with the variations of enzymatic activities. For example, under drought stress condition, the CAT activity in grafted plants increased significantly, whereas expression level decreased in leaves and did not change in roots. The lack of correlation between expression level and enzymatic activity may be due to different gene subtypes in plant cells. A similar result has been found in Citrus [74]. In a word, this indicated that in the grafted plants, the three antioxidant enzymes efficiently detoxified the amounts of H_2_O_2_, promoted the scavenging of ROS, and then reduced the lipid peroxidation induced by drought stress in grapevine. In this way, grafted plants developed a better antioxidant system to scavenge harmful ROS which was clearly confirmed by remarkably lower contents of H_2_O_2_ and MDA in grafted plants. A similar phenomenon was also observed in other research [75–77].

## Conclusions

**Fig. 12:**
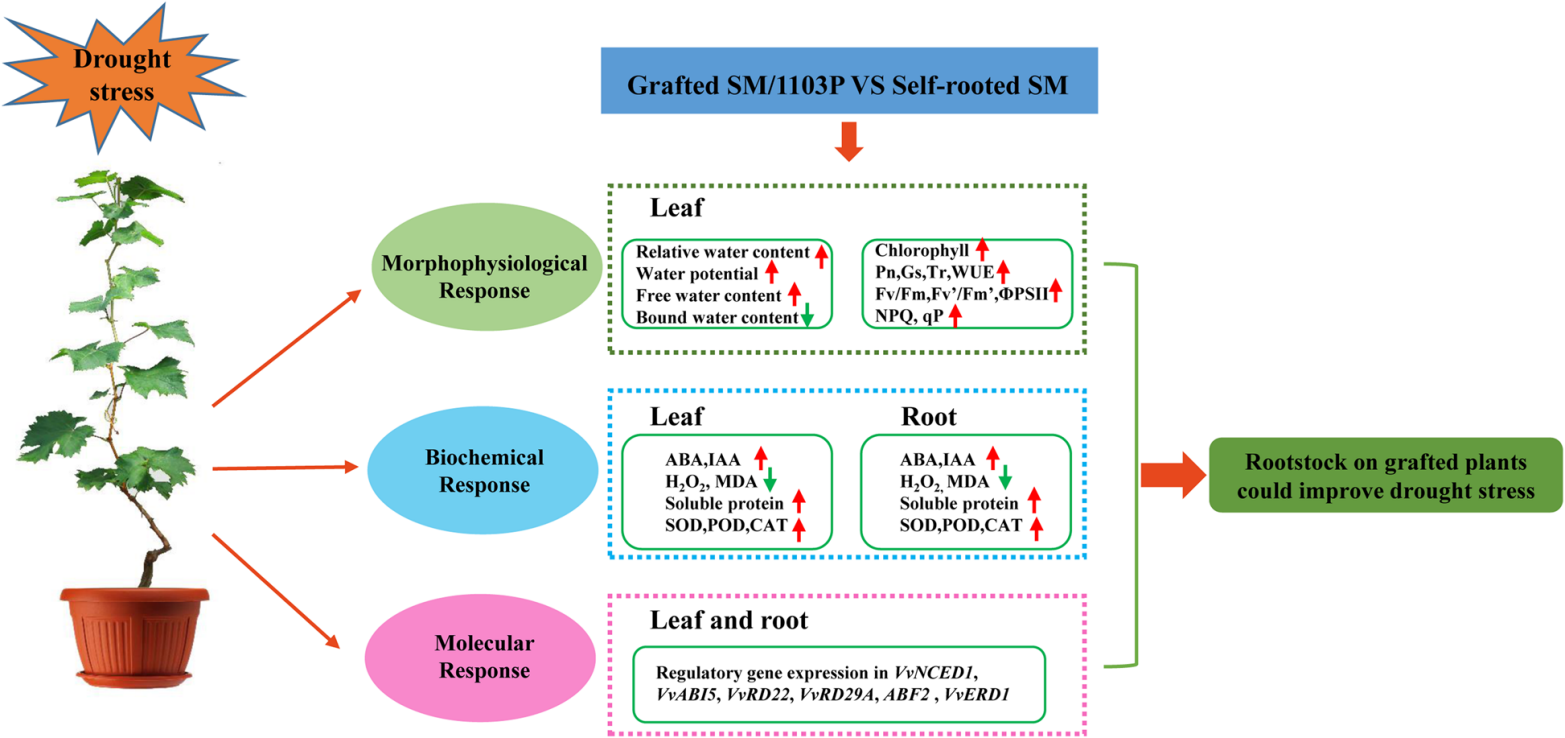
A schematic model of the grafted grapevines and self-rooted SM in response to drought stress

In summary, compared with self-rooted SM, the grafted grapevine could ameliorate drought tolerance by maintaining higher RWC, water potential and free water content of leaves, modulating photosynthetic performance and levels of hormones, as well as alleviating the accumulation of ROS, maintaining redox homeostasis and activating stress-responsive gene expression (Fig. 12). Our study provides valuable resources for further investigations on grafting mediated drought regulation mechanisms, and will accelerate the potential application of grafting technology in improving drought resistance on grapes.

## Materials and methods

### Plant materials

Two-year-old self-rooted rootstock 1103P (*V. berlandieri* × *V. rupestris*), self-rooted table grape cultivar ‘SM’ (*V. labrusca* × *V. vinifera*), and grafted grape (SM shoot/1103P root) were grown in the greenhouse of the College of Horticulture (25/20 ± 2 °C day/night, 16/8 h photoperiod, and 75% relative humidity), Gansu Agricultural University, located in Lanzhou, China. All of the plant materials were supplied from Zhichang Grape Research Institute, Shandong Province of China, and grown in a 12 L ceramic pot (containing peat moss, perlite and vermiculite: 3:1:1, v/v/v). Before treatment, the grapevines were regularly watered to maintain optimal soil moisture.

### Drought treatment

A total of 60 plants from each grape material were randomly divided into two groups: plants grown under well-watered condition and plants grown under drought stress condition, and 30 plants in each group. For the well-watered samples, the soil volumetric moisture content was maintained approximately 60%, whereas for drought stress samples, the volumetric water content of soil was progressively reduced from 60 to 20% and was maintained at the level for 40 d (Supplementary Fig. 2). Soil moisture content was measured with a Hand-held soil moisture meter (Handi-trase, Zealquest Scientific Technology Co., Ltd). During the drought treatment, the required amount of water was replenished every day at 18:00 to maintain the established soil water content in all pots. The whole drought stress experiment lasted for 40 d. The samples of leaves of similar size were collected from the third to fifth leaves (counting from the tip of each branch), and roots were separated from the soil, washed free of soil using deionized water. Samples were harvested at 0, 20 and 40 d after stress imposition, frozen in liquid nitrogen and stored in a refrigerator at − 80 °C. Physiological, biochemical indexes and gene expression were tested at corresponding days. The experiments were performed in triplicate and independently validated with 3 biological replicates.

### Measurement of relative water content and leaf water potential

Relative water content (RWC) and leaf water potential (Ψ) were measured by selecting the node of 7–9th from the base of grapevine leaves. For the measurement of RWC, grapevine leaves with drought treatment and well-watered were collected, stored in dark containers and quickly brought back to the laboratory to record the fresh weight (FW). Then the leaves were immersed in distilled water for 24 h at 4 °C in darkness, following which the turgid weight (TW) was obtained. Finally, the samples were dried at 105 °C for 30 min and at 80 °C until constant weight, and the dry weight (DW) was determined. Relative water content (RWC) was calculated according to the formula: RWC (%) = (FW-DW) / (TW-DW) × 100%.

The predawn leaf water potential was determined using a PSYPRO water potential system (Wescor Inc., USA). The total and free water content was measured according to the reported method [78], and the bound water was calculated by the formula: bound water content (%) = Total water content (%)-free water content (%).

### Measurement of chlorophyll content

To measure the content of chlorophyll, 0.2 g fresh leaves were removed from each grape material, cut into 2 mm pieces and extracted with 10 mL of 80% (v/v) acetone in darkness until the leaves turned white. The absorbance of the extract was monitored with a spectrophotometer (Shimadzu, UV-1780, Japan) at wavelengths 470, 646 and 663 nm, respectively. The content of chlorophyll was calculated according to the reported method [79].

### Measurement of photosynthetic parameters and chlorophyll fluorescence parameters

Plant leaf photosynthetic parameters, including net photosynthetic rate (Pn), stomatal conductance (Gs), transpiration rate (Tr) were measured using a portable photosynthesis system (LI-6400, LI-COR, USA). The determination parameters were set as follows: the saturating light was set at 1000 μmol·m^− 2^·s^− 1^, airflow speed was 500 μmol·s^− 1^ and the ambient concentration CO_2_ in the leaf chamber was maintained at 400 μmol·mol^− 1^. Measurements were performed on sunny days between 9:30 to 11:30 a.m. For each measurement, the 4th fully expanded leaves exhibiting uniform growth plants at the same positions were used.

The chlorophyll fluorescence parameters of grape leaves were measured using a chlorophyll fluorescence imaging system (Technologica, UK). Selecting the 7th to 10th leaves from the base for adapting to darkness about 30 minutes, and initial fluorescence (Fo) was measured. Then, the maximal fluorescence (Fm) was measured by the saturation light pulse (5000 μmol m^− 2^·s^− 1^) when all photosystem II reaction centers were closed. The maximum quantum efficiency of photosystem II (Fv/Fm), excitation energy capture efficiency of open PSII reaction centers under light (Fv’/Fm′), the actual quantum yield of photosynthesis (ΦPSII), the nonphotochemical quenching coefficient (NPQ), the photochemical quenching coefficient (qP), and Excitation pressure of PSII (1-qP) were also measured and calculated by the chlorophyll fluorescence imaging system. Three independent biological replicates for each treatment were performed.

### Measurement of endogenous hormone content

The ABA and IAA content was performed by HPLC according to the method as previously described with slight modifications [80]. Briefly, 1.0 g samples were quickly and fully ground in liquid nitrogen and 5 mL 80% chromatographic methanol was added to the powder. Then, the extract was centrifuged at 4 °C 12,000 rpm for 10 min. Using a rotary evaporator evaporated the extract under 38 °C (1300 rpm/min) to obtain 2 mL of sample concentrate. Afterwards, the dry samples were redissolved in 2 mL 50% chromatographic methanol (v/v, methanol/water), and analyzed by HPLC on a Aglient Zorbax SB-C18 column (5 μm, 250 mm × 4.6 mm) at 30 °C column temperature.

### Measurement of H_2_O_2_, MDA, antioxidant enzymes and soluble protein

The content of hydrogen peroxide (H_2_O_2_) and malondialdehyde (MDA), the activities of superoxide dismutase (SOD), peroxidase (POD) and catalase (CAT) were determined using the corresponding commercial assay kits (Keming, Suzhou, China). The content of soluble protein was determined according to standard procedures [79].

### Quantitative real-time PCR

The total RNA from leaves and roots was isolated with E.Z.N.A.Plant RNA Kit (Omega, USA). The first-strand cDNA was synthesized from 1 μg total RNA in a 20 μL reaction using Evo M-MLV RT Kit II (Accurate Biology, Hunan China), and qRT-PCR was conducted on a LightCycle 96 Real-Time PCR System (Roche, USA) using SYBR Green premix pro Taq (Accurate Biology, Hunan China), as described in the manufacturer’s protocol. The grape actin gene (GenBank accession no. XM_002282480.4) was used as a reference, and the primers used for qRT-PCR were listed in Table S1. Three biological replicates were analyzed and the data were analyzed using the the 2^− ΔΔCT^ method [81].

### Statistical analysis

Expression levels of related genes in leaves and roots of grapevine were analyzed by one-way ANOVA. RWC, Ψ, total water content, free and bound water content, chlorophyll, carotenoid, photosynthetic parameters, chlorophyll fluorescence, the level of ABA and IAA, H_2_O_2,_ MDA, soluble protein, and activities of antioxidant enzyme were examined by two-way ANOVA. All the data for each treatment were the average values of at least three replicates, and statistical analysis was conducted by Duncan’s multiple range test at *P < 0.05* using SPASS25.0 software. Principal component analysis (PCA) and all figures were performed with Origin2021 software (OriginLab, USA).

## Supplementary Information

**Additional file 1: Supplementary Fig. 1** Principal component analysis of the internal quality parameters of self-rooted and grafted vines. **Supplementary Fig. 2** Soil volumetric moisture content of well-watered and drought stress in self-rooted and grafted vines.

**Additional file 2: Table S1.** Primers used for RT-PCR.

## Data Availability

All data analyzed in this study are included within the article and attached to the supplementary information files.

## Abbreviations

SM: Shine Muscat
SM/1103P: SM shoot/1103P root
SOD: superoxide dismutase
POD: peroxidase
CAT: catalase
ABA: abscisic acid
IAA: indoleacetic acid
RWC: relative water content
Ψ: water potential
FW: fresh weight
TW: turgid weight
DW: dry weight
Pn: net photosynthetic rate
Gs: stomatal conductance
Tr: transpiration rate
Fo: initial fluorescence
Fm: maximal fluorescence
Fv/Fm: the maximum quantum efficiency of photosystem II
Fv’/Fm′: excitation energy capture efficiency of open PSII reaction centers under light
ΦPSII: the actual quantum yield of photosynthesis
NPQ: the nonphotochemical quenching coefficient
qP: the photochemical quenching coefficient
1-qP: excitation pressure of PSII
H_2_O_2_: hydrogen peroxide
MDA: malondialdehyde
PCA: principal component analysis
*NCED1*: 9-cis-epoxycarotenoid dioxygenase 1
*ABI5*: ABA insensitive 5
*b**Z**I**P*: basic leucine zipper
*ERD1*: early responsive to dehydration stress 1
PCs: principle components
CK: well-watered
DS: drought stress
ROS: oxygen species
O_2_^−^: superoxide anion

## References

[1] Zhu JK (2002). Salt and drought stress signal transduction in plants. Annu Rev Plant Biol.

[2] Osmolovskaya N, Shumilina J, Kim A, Didio A, Grishina T, Bilova T (2018). Methodology of drought stress research: experimental setup and physiological characterization. Int J Mol Sci.

[3] Gupta A, Rico-Medina A, Caño-Delgado AI (2020). The physiology of plant responses to drought. Science..

[4] Skirycz A, Inze D (2010). More from less: plant growth under limited water. Curr Opin Biotechnol.

[5] Fanizza G, Ricciardi L (2015). Influence of drought stress on shoot, leaf growth, leaf water potential, stomatal resistance in wine grape genotypes (*Vitis vinifera* L.). Vitis..

[6] Siddique Z, Jan S, Imadi SR, Gul A, Ahmad P (2016). Drought stress and photosynthesis in plants. Water stress and crop plants: a sustainable approach.

[7] Siddique Z, Jan S, Imadi SR, Gul A, Ahmad P (2016). Drought stress and photosynthesis in plants.

[8] Tsugane K, Kobayashi K, Niwa Y, Ohba Y, Wada K, Kobayashi H (1999). A recessive arabidopsis mutant that grows photoautotrophically under salt stress shows enhanced active oxygen detoxification. Plant Cell.

[9] Li Y, Zhao HX, Duan BL, Korpelainen H, Li CY (2011). Effect of drought and ABA on growth, photosynthesis and antioxidant system of Cotinus coggygria seedlings under two different light condition. Environ Exp Bot.

[10] Sarker U, Oba S (2018). Drought stress effects on growth, ROS markers, compatible solutes, Phenolics, flavonoids, and antioxidant activity in Amaranthus tricolor. Appl Biochem Biotech.

[11] Hoffman L, Dacosta M, Ebdon JS, Zhao J (2012). Effects of drought preconditioning on freezing tolerance of perennial ryegrass. Environ Exp Bot.

[12] Salazar-Parra C, Aguirreolea J, Sánchez-Díaz M, Irigoyen JJ, Morales F (2012). Climate change (elevated CO2, elevated temperature and moderate drought) triggers the antioxidant enzymes' response of grapevine cv. Tempranillo, avoiding oxidative damage. Physiol Plant.

[13] Sheikh-Mohamadi MH, Etemadi N, Arab MM, Aalifar M, Arab M (2018). Physiological and ascorbate-glutathione pathway-related genes responses under drought and heat stress in crested wheatgrass. Sci Hortic.

[14] Per TS, Khan NA, Reddy PS, Masood A, Hasanuzzaman M, Khan MIR (2017). Approaches in modulating proline metabolism in plants for salt and drought stress tolerance: phytohormones, mineral nutrients and transgenics. Plant Physiol Biochem.

[15] Kuromori T, Seo M, Shinozaki K. ABA transport and plant water stress responses. Trends Plant Sci. 2018;23(6):513–22. 10.1016/j.tplants.2018.04.001 29731225

[16] Yoshida T, Obata T, Feil R, Lunn JE, Fujita Y, Kazuko YS, Fernie AR (2019). The role of Abscisic acid signaling in maintaining the metabolic balance required for Arabidopsis growth under non-stress condition. Plant Cell.

[17] Bletsos FA, Olympios CM (2008). Rootstocks and grafting of tomatoes, peppers and eggplants for soil-borne disease resistance, improved yield and quality. Eur J Plant Sci Biotechnol.

[18] Mudge K, Janick J, Scofield S, Goldschmidt EE (2009). A history of grafting. Hortic Rev.

[19] Lee JM, Kubota C, Tsao SJ, Bie Z, Echevarria PH, Morra L (2010). Current status of vegetable grafting: diffusion, grafting techniques, automation. Sci Hortic.

[20] Warschefsky EJ, Klein LL, Frank MH, Chitwood DH, Londo JP, von Wettberg EJB (2016). Rootstocks: diversity, domestication, and impacts on shoot phenotypes. Trends Plant Sci.

[21] Granett J, Walker MA, Kocsis L, Omer AD (2001). Biology and management of grape phylloxera. Annu Rev Entomol.

[22] Li Z, Elisa M, Landry R, Nathalie O, Gregory AG. The influence of grapevine rootstocks on scion growth and drought resistance. Theoretical and experimental. Plant Physiol. 2016;28(2):143–57.

[23] Davis AR, Perkins-Veazie P, Hassell R, Levi A, King SR, Zhang X (2008). Grafting effects on vegetable quality. HortSci.

[24] Paranychianakis NV, Aggelides S, Angelakis AN (2004). Influence of rootstock, irrigation level and recycled water on growth and yield of Soultanina grapevines. Agric Water Manag.

[25] Ollat N, Tandonnet JP, Lafontaine M, Schultz H (2001). Short and long term effects of three rootstocks on cabernet sauvignon vine behaviour and wine quality. Acta Hortic.

[26] Keller M, Mills LJ, Harbertson JF (2012). Rootstock effects on deficit-irrigated winegrapes in a dry climate: vigor, yield formation, and fruit ripening. Am J Enol Vitic.

[27] Corso M, Bonghi C (2014). Grapevine rootstock effects on abiotic stress tolerance. Plant Sci Today.

[28] Serra I, Strever A, Myburgh PA, Deloire A (2014). Review: the interaction between rootstocks and cultivars (*Vitis vinifera* L.) to enhance drought tolerance in grapevine. Aust J Grape Wine R.

[29] Stoll M, Loveys B, Dry P (2000). Hormonal changes induced by partial rootzone drying of irrigated grapevine. J Exp Bot.

[30] Soar CJ, Dry PR, Loveys BR (2006). Scion photosynthesis and leaf gas exchange in *Vitis vinifera* L. vv. Shiraz: mediation of rootstock effects via xylem sap ABA. Aust J Grape Wine R.

[31] Koundouras S, Tsialtas IT, Zioziou E, Nikolaou N (2008). Rootstock effects on the adaptive strategies of grapevine (*Vitis vinifera* L. cv. Cabernet–sauvignon) under contrasting water status: leaf physiological and structural responses. Agric Ecosyst Environ.

[32] Dodd IC (2013). Abscisic acid and stomatal closure: a hydraulic conductance conundrum?. New Phytol.

[33] McAdam SAM, Brodribb TJ (2015). The evolution of mechanisms driving the stomatal response to vapor pressure deficit. Plant Physiol.

[34] Marguerit E, Brendel O, Lebon E, Leeuwen CV, Ollat N (2012). Rootstock control of scion transpiration and its acclimation to water deficit are controlled by different genes. New Phytol.

[35] Rossdeutsch L, Edwards E, Cookson SJ, Barrieu F, Gambetta GA, Delrot S (2016). ABA-mediated responses to water deficit separate grapevine genotypes by their genetic background. BMC Plant Biol.

[36] Berdeja M, Nicolas P, Kappel C, Dai ZW, Hilbert G, Peccoux A (2015). Water limitation and rootstock genotype interact to alter grape berry metabolism through transcriptome reprogramming. Hortic Res.

[37] Wang WN, Min Z, Wu JR, Liu BC, Xu XL, Fang YL (2021). Physiological and transcriptomic analysis of cabernet Sauvginon (*Vitis vinifera* L.) reveals the alleviating effect of exogenous strigolactones on the response of grapevine to drought stress. Plant Physiol Biochem.

[38] Albacete A, Andújar C, Dodd I, Giuffrida F, Hichri I, Lutts S (2015). Rootstock-mediated variation in tomato vegetative growth under drought, salinity and soil impedance stresses. Acta Hortic.

[39] Li CH, Li YS, Bai LQ, He CX, Yu XC (2016). Dynamic expression of miRNAs and their targets in the response to drought stress of grafted cucumber seedlings. Hortic Plant J.

[40] Anjum SA, Xie XY, Wang LC, Saleem MF, Man C, Lei W (2011). Morphological, physiological and biochemical responses of plants to drought stress. Afr J Agric Res.

[41] Kadioglu A, Saruhan N, Sağlam A, Terzi R, Acet T (2011). Exogenous salicylic acid alleviates effects of long term drought stress and delays leaf rolling by inducing antioxidant system. Plant Growth Regul.

[42] Kramer PJ (1983). Water relations of plants.

[43] Martínez-Vilalta J, Garcia-Forner N (2017). Water potential regulation, stomatal behaviour and hydraulic transport under drought: deconstructing the iso/anisohydric concept. Plant Cell Environ.

[44] Bota J, Tomás M, Flexas J, Medrano H, Escalona JM (2016). Differences among grapevine cultivars in their stomatal behavior and water use efficiency under progressive water stress. Agr Water Manage.

[45] Meng JF, Xu TF, Wang ZZ, Fang YL, Xi ZM, Zhang ZW (2014). The ameliorative effects of exogenous melatonin on grape cuttings under water-deficient stress: antioxidant metabolites, leaf anatomy, and chloroplast morphology. J Pineal Res.

[46] Min Z, Li RY, Chen L, Zhang Y, Li ZY, Liu M (2018). Alleviation of drought stress in grapevine by foliar-applied strigolactones. Plant Physiol Biochem.

[47] Zlatev Z (2009). Drought–induced changes in chlorophyll fluorescence of young wheat plants. Biotechnol Biotech Eq.

[48] Cornic G (2000). Drought stress inhibits photosynthesis by decreasing stomatal aperture-not by affecting ATP synthesis. Trends Plant Sci.

[49] Liang B, Ma C, Zhang Z, Wei Z, Gao T, Zhao Q (2018). Long-term exogenous application of melatonin improves nutrient uptake fluxes in apple plants under moderate drought stress. Environ Exp Bot.

[50] Liu BH, Cheng L, Liang D, Zou YJ, Ma FW (2012). Growth, gas exchange, water–use efficiency, and carbon isotope composition of ‘Gale gala’ apple trees grafted onto 9 wild Chinese rootstocks in response to drought stress. Photosynthetica..

[51] Chen Y, Sun XZ, Zheng CS, Zhang S, Yang JH (2018). Grafting onto Artemisia annua improves drought tolerance in chrysanthemum by enhancing photosynthetic capacity. Hortic Plant J.

[52] Chen K, Li GJ, Bressan RA, Song CP, Zhu JK, Zhao Y (2020). Abscisic acid dynamics, signaling, and functions in plants. J Integr Plant Biol.

[53] Chen XX, Ding YL, Yang YQ, Song CP, Wang BS, Yang SH (2021). Protein kinases in plant responses to drought, salt, and cold stress. J Integr Plant Biol.

[54] Bano A, Ullah F, Nosheen A (2012). Role of abscisic acid and drought stress on the activities of antioxidant enzymes in wheat. Plant Soil Environ.

[55] Brodribb TJ, McAdam SAM (2013). Abscisic acid mediates a divergence in the drought response of two conifers. Plant Physiol.

[56] Teale WD, Paponov IA, Palme K (2006). Auxin in action: signalling, transport and the control of plant growth and development. Nat Rev Mol Cell Biol.

[57] Spaepen S, Vanderleyden J, Remans R (2007). Indole-3-acetic acid in microbial and microorganism-plant signaling. FEMS Microbiol Rev.

[58] Spaepen S, Vanderleyden J (2011). Auxin and plant-microbe interactions. Csh Perspect Biol.

[59] Zhao MR, Han YY, Feng YN, Li F, Wang W (2012). Expansins are involved in cell growth mediated by abscisic acid and indole-3-acetic acid under drought stress in wheat. Plant Cell Rep.

[60] Mezer M, Turska-Taraska A, Kaczmarek Z, Glowacka K, Swarcewicz B, Rorat T (2014). Differential physiological and molecular response of barley genotypes to water deficit. Plant Physiol Biochem.

[61] Tu MX, Wang XH, Feng TY, Sun XM, Wang YQ, Huang L (2016). Expression of a grape (*Vitis vinifera*) bZIP transcription factor, VlbZIP36, in Arabidopsis thaliana confers tolerance of drought stress during seed germination and seedling establishment. Plant Sci.

[62] Tu MX, Wang XH, Huang L, Guo RR, Zhang HJ, Cai JS (2016). Expression of a grape bZIP transcription factor, *VqbZIP39*, in transgenic Arabidopsis thaliana confers tolerance of multiple abiotic stresses. Plant Cell Tiss Org.

[63] Skubacz A, Daszkowska-Golec A, Szarejko L (2016). The role and regulation of ABI5 (ABA-insensitive 5) in plant development, abiotic stress responses and Phytohormone crosstalk. Front Plant Sci.

[64] Mittler R (2002). Oxidative stress, antioxidants and stress tolerance. Trends Plant Sci.

[65] Miller G, Suzuki N, Ciftci-Yilmaz S, Mittler R (2010). Reactive oxygen species homeostasis and signalling during drought and salinity stresses. Plant Cell Environ.

[66] Liu JJ, Li JQ, Su XH, Xia ZL (2014). Grafting improves drought tolerance by regulating antioxidant enzyme activities and stress-responsive gene expression in tobacco. Environ Exp Bot.

[67] Choudhury FK, Rivero RM, Blumwald E, Mittler R (2017). Reactive oxygen species, abiotic stress and stress combination. Plant J.

[68] Scandalios JG (1993). Oxygen stress and superoxide Dismutases. Plant Physiol.

[69] Ma QQ, Wang W, Li YH, Li DQ, Zou Q (2006). Alleviation of photoinhibition in drought-stressed wheat (*Triticum aestivum*) by foliar-applied glycinebetaine. J Plant Physiol.

[70] Rahimi M, Kordrostami M, Maleki M, ModaresKia M (2016). Investigating the effect of drought stress on expression of *WRKY1* and *EREBP1* genes and antioxidant enzyme activities in lemon balm (*Melissa Officinalis* L.). Biotech..

[71] Gunes A, Pilbeam DJ, Inal A, Coban S (2008). Influence of silicon on sunflower cultivars under drought stress, I: growth, antioxidant mechanisms, and lipid peroxidation. Commun Soil Sci Plant.

[72] Tohidi-Moghadam HR, Shirani-Rad AH, Nour-Mohammadi G, Habibi D, Mashhadi-Akbar-Boojar M (2009). Effect of super absorbent application on antioxidant enzyme activities in canola (*Brassica napus* L.) cultivars under water stress condition. Am J Agric Biol Sci.

[73] Jin SH, Li XQ, Jia XL (2011). Genotypic differences in the responses of gas exchange, chlorophyll fluorescence, and antioxidant enzymes to aluminum stress in Festuca arundinacea. Russ J Plant Physiol.

[74] Balfagón D, Terán F, Oliveira TDRD, Santa-Catarina C, Gómez-Cadenas A (2021). Citrus rootstocks modify scion antioxidant system under drought and heat stress combination. Plant Cell Rep.

[75] Li YH, Liu YJ, Xu XL, Jin M, An LZ, Zhang H (2012). Effect of 24-epibrassinolide on drought stress-induced changes in Chorispora bungeana. Biol Plant.

[76] Radhakrishnan R, Lee I (2013). Spermine promotes acclimation to osmotic stress by modifying antioxidant, abscisic acid, and jasmonic acid signals in soybean. J Plant Growth Regul.

[77] Talaat NB, Shawky BT, Ibrahim AS (2015). Alleviation of drought-induced oxidative stress in maize (*Zea mays* L.) plants by dual application of 24-epibrassinolide and spermine. Environ Exp Bot.

[78] Wang BL. Effects of drought stress on water physiology and expression of *AQPs* Geans to *Malus zumi*. Gansu Agric Univ. 2018.

[79] Gao JF (2006). Experimental guidance for plant physiology.

[80] Li WF, Mao J, Li XW, Su J, Dawuda MM, Ma ZH (2018). Effects of CEPA and 1-MCP on flower bud differentiation of apple cv. 'Nagafu no.2′ grafted on different rootstocks. J Plant Growth Regul.

[81] Livak KJ, Schmittgen TD (2001). Analysis of relative gene expression data using real-time quantitative PCR. Methods..

