# Supplementary material for: Physiological, biochemical and molecular responses associated with drought tolerance in grafted grapevine
Source: BMC Plant Biol. 2023 Feb 23;23:110. doi: 10.1186/s12870-023-04109-x (PMC9948381; doi:10.1186/s12870-023-04109-x)
Supplement: Supplementary file 1 — Additional file 1: Supplementary Fig. 1 Principal component analysis of the internal quality parameters of self-rooted and grafted vines. Supplementary Fig. 2 Soil volumetric moisture content of well-watered and drought stress in self-rooted and grafted vines. [file 12870_2023_4109_MOESM1_ESM.docx]

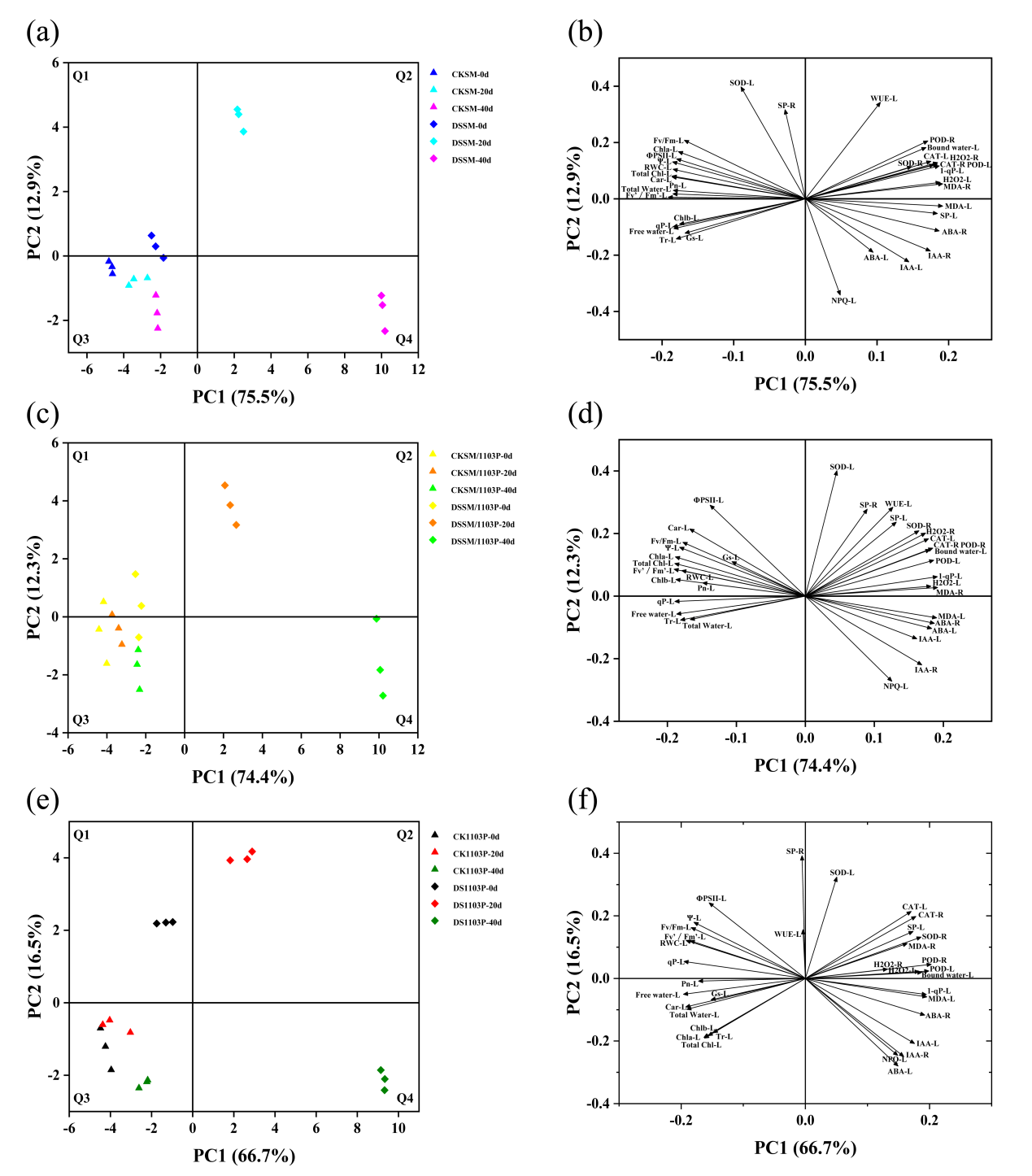


**Supplementary fig.1** Principal component analysis of the internal quality parameters of self-rooted and grafted vines.(a) Score plot of SM; (b) loading plot of SM; (c) Score plot of SM/1103P; (d) loading plot of SM/1103P; (e) Score plot of 1103P; (f) loading plot of 1103P. “△” represented well-watered samples and“◇” represented drought stress samples.


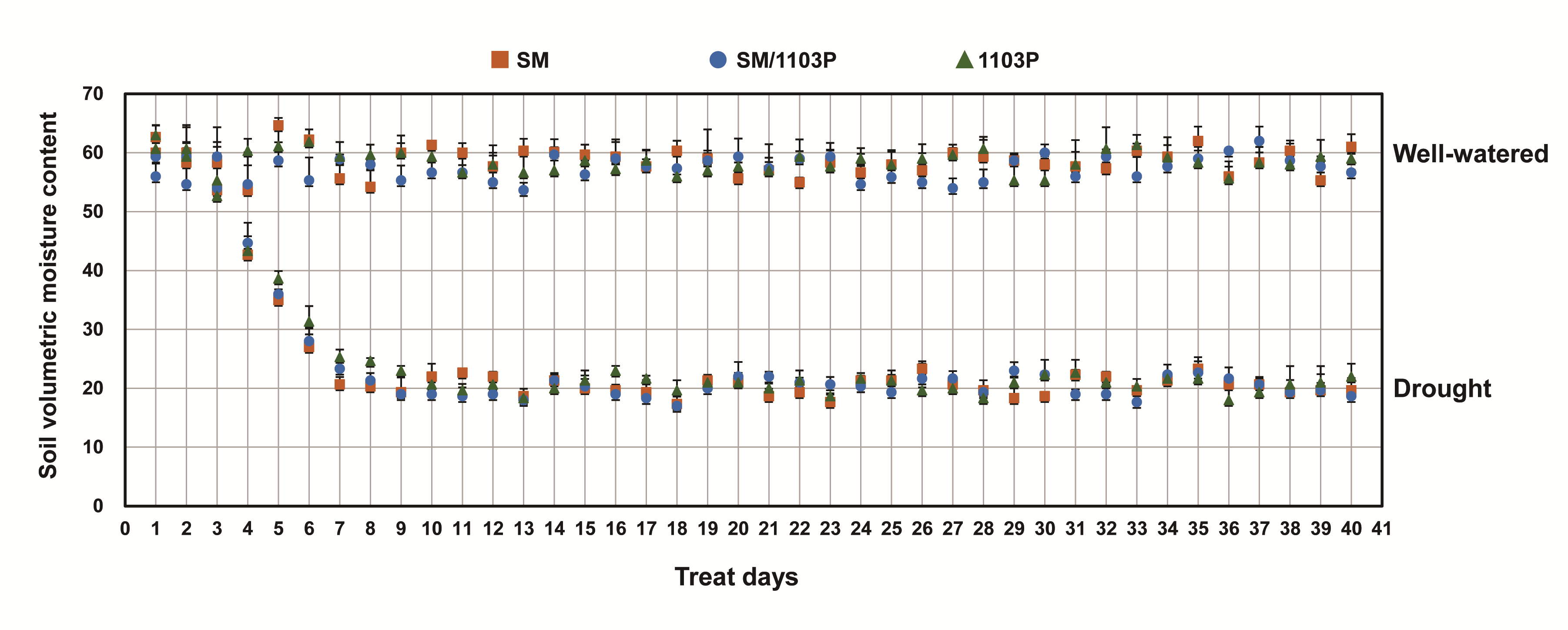


**Supplementary fig.2** Soil volumetric moisture content of well-watered and drought stress in self-rooted and grafted vines. Well-watered plants were maintained at 60% of soil volumetric content, and drought stress was progressively reduced until 20% of soil volumetric content and maintained for 40 days. The values are the means ± SD (n = 6).
