# Supplementary material for: Physiological, biochemical and molecular responses associated with drought tolerance in grafted grapevine
Source: BMC Plant Biol. 2023 Feb 23;23:110. doi: 10.1186/s12870-023-04109-x (PMC9948381; doi:10.1186/s12870-023-04109-x)
Supplement: Supplementary file 2 — Additional file 2: Table S1. Primers used for RT-PCR. [file 12870_2023_4109_MOESM2_ESM.doc]

**Table S1 Primers used for RT-PCR**

| **Primer name** | **Primers(5'-3')** |
| --- | --- |
| RT-VvCu/ZnSOD-F  RT- VvCu/ZnSOD-R | GGTGAAGGCTGTTGCTGTTCTTAAC  CCAGTCACTGTAGTCGAACCATCTC |
| RT-VvPOD4-F  RT-VvPOD4-R | CGACGACACCTCTTCCTTCACTG  CCTCCAAGAATCACGACCGAGTC |
| RT-VvCAT1-F  RT-VvCAT1-R | GCTATCTTGACTGGAAGGCGTGAA  GATGCTGCGGATCTCATAGGTGAC |
| RT-VvNCED1-F  RT-VvNCED1-R | GGTTCCAGATCAGCAGGTTGTGTT  GCATACCAGAAGCGTCAGTAGCATT |
| RT-VvABI5-F  RT-VvABI5-R | ACTGTGGATGAGGTCTGGTCTGAA  CGTACTGATGTTGCTGCTGATGCT |
| RT-VvRD22-F  RT-VvRD22-R | ATACAGACACATCGGCATGGAACC  CTCAGGAAGGAAATGGCAGATTGGA |
| RT-VvRD29A-F  RT-VvRD29A-R | GGTGATGGTGGTGAAGAAGTGGTAA  AACGGTTGTTCTGGCTTATCCTGTA |
| RT-VvABF2-F  RT-VvABF2-R | AACGGTCATCACAGCAGCAACTC  CGCCACCATGAAGCACACTACTC |
| RT-VvERD1-F  RT-VvERD1-R | TGTGAAGTCGGTGAACAATCTCGTT  CGCATCAACAGCATCAACCATTCC |
